# Sexual Pleasure Matters – and How to Define and Assess It Too. A Conceptual Framework of Sexual Pleasure and the Sexual Response

**DOI:** 10.1080/19317611.2023.2212663

**Published:** 2023-06-12

**Authors:** Marlene Werner, Michèle Borgmann, Ellen Laan

**Affiliations:** a Department of Sexology and Psychosomatic Obstetrics and Gynaecology, Amsterdam University Medical Center, University of Amsterdam, Amsterdam, The Netherlands; b Department of Health Psychology and Behavioral Medicine, Institute of Psychology, University of Bern, Bern, Switzerland

**Keywords:** Sexual pleasure, sexual response, state and trait, taxonomy, psychometrics

## Abstract

**Objective:**

Sexual pleasure is central to current understandings of sexual function, health, and wellbeing. In this article, we suggest that we lack a sufficiently specific, yet encompassing, definition of sexual pleasure and that we therefore lack comprehensive assessments of sexual pleasure. We introduce a definition of sexual pleasure and position it centrally in an adapted framework of the sexual response. In the framework, we include a taxonomy of rewards which can be retrieved from sex and thereby aim to capture the multifaceted nature of sexual pleasure.

**Methods/Results:**

Through narrative review, we arrive at the definition, framework, and taxonomy by integrating theories of sexual motivation and response with the literature on sexual pleasure and basic rewards. We position this literature within theories of affect and personality which allows us to differentiate between the experience of and the tendency to experience sexual pleasure (i.e., state versus trait sexual pleasure). We discuss how this conceptualization of sexual pleasure could be reflected in self-report assessments to quantitatively assess sexual pleasure.

**Conclusions:**

The framework may aid to understand the role of the diverse facets of sexual pleasure in sexual function, health, and wellbeing and contribute to giving sexual pleasure the center position it deserves in sex research and therapy.

At the beginning of the 21st century, the World Health Organization (WHO, 2002) included pleasure as a component in their definition of sexual health. This definition further championed the sex-positive perspective in sex research, practice, and advocacy (Arakawa et al., 2013; Fine, 1988; Kleinplatz et al., 2009; Philpott et al., 2006), assisted by efforts from the Global Advisory Board for Sexual Health and Wellbeing (GAB) and the World Association of Sexual Health (WAS). In this article, we aim to tackle a crucial puzzle piece in this sex-positive endeavor. We suggest that we should build on currently available definitions of sexual pleasure to make them more specific yet comprehensive and that such specification allows us to create more valid assessments of sexual pleasure. In this article, we have three main aims: (1) to provide a definition of sexual pleasure within a conceptual framework that positions pleasure within theories of the sexual response specifically, and within theories of *states* and *traits* more generally, (2) to describe a taxonomy of rewards which induce pleasure during sexual activity and which allows for a multifaceted perspective on sexual pleasure, and (3) to discuss how the definition, framework, and taxonomy interrelate with specifically structured assessments and research opportunities of (state and trait) sexual pleasure.

## Aim 1: Where does sexual pleasure figure in the sexual response? From sex drive to desire for rewarding sex

### Available definitions of sexual pleasure

Sexual pleasure has regularly hidden from view during the advent of sexology as a science (Clark, 2006; Jones, 2019). A few bold theorists revived the discussion of sexual pleasure and aimed to understand and advocate for this historically contentious concept (Clark, 2006; Ford et al., 2019). These contributions served as an important foundation for the WAS Declaration on Sexual Pleasure, have informed our understanding of sexual pleasure and its definition (Ford et al., 2019, 2021), and their descendants were essential to create the base from which we can endeavor further. We suggest that we can build on our currently available definitions of sexual pleasure to become more specific yet comprehensive. Table 1 provides an overview of existing definitions of sexual pleasure.

**Table 1.** Definitions of Sexual Pleasure.

Source	Definition
Guggino and Ponzetti (1997) cited and adapted in Katz and Schneider (2015, p. 453)	“[Sexual] Pleasure includes positive feelings of satisfaction, [excitement,] love, and romance.” [information in brackets added to this sentence from other parts of the same article]
Abramson and Pinkerton (2002, p. 8)	“Sexual pleasure consists of those positively valued feelings induced by sexual stimuli. Notice that this conceptualization encompasses a broad range of sexual pleasures, from the soothing sensations of sensual massage, to the explosion of feeling that accompanies orgasm.”
Horne and Zimmer-Gembeck (2006, p. 126)	“Sexual pleasure is defined as a sense of well-being derived from the experience of being sexual and, as such, is an essential component of sexual subjectivity.”
De la Garza-Mercer (2007, p. 108)	“[…] for the purpose of our discussion and for the sake of simplicity, *sexual pleasure* primarily refers to the positive physical and subjective sensation and emotional experience resulting from stimulation of the genitals, breasts, and other erogenous zones. In this way, “sexual pleasure” encompasses a narrow range of direct behaviors that set in motion the aforementioned thoughts and behaviors that are more distally related to the fundamental factor of genital stimulation.”
Philpott et al. (2006, p. 23)	“Sexual pleasure is the physical and/or psychological satisfaction and enjoyment one derives from any erotic interaction.”
Higgins and Hirsch (2008) cited in Anderson (2013, p. 209)	No definition established. However, they propose five components of sexual pleasure: “[…] something that feels good, spontaneity and flow, closeness, partner’s pleasure, and eroticization of safety. The diversity of these components highlight the range of physical, emotional, and social factors that are at play in the experience of sexual pleasure; since sexual pleasure is often (although certainly not always) experienced with another person, interpersonal experiences are often involved in pleasure.”
Züricher Institut für klinische Sexologie und Sexualtherapie (ZISS, 2013, p. 9)	“Sexual pleasure is the ability to enjoy sexual arousal. […] since the brain and body form a functional unity, an improvement of the arousal function will directly affect sexual pleasure and, consequently, the ability to orgasm.”
Global Advisory Board for Sexual Health and Wellbeing (GAB, 2016)	“Sexual pleasure is the physical and/or psychological satisfaction and enjoyment derived from solitary or shared erotic experiences, including thoughts, dreams and autoeroticism.”
Goldey et al. (2016, p. 2148)	“[…] sexual pleasure is highly multifaceted, encompassing physical experiences (e.g., sensory stimulation), cognitive experiences (e.g., getting outside the self), and emotional experiences (e.g., trust), as well as experiences that challenge mind-body dualisms (e.g., autonomy, which could facilitate intense genital pleasure and unique emotional experiences).”
Fahs and Plante (2017, p. 33)	“Analysis revealed four themes in women’s descriptions of good, happy and joyous sex: (1) Physical pleasure, wanting and orgasm; (2) Emotional connection and relationship satisfaction; (3) Comfort and naturalness; (4) Control over sexual scripts.”
Fileborn et al. (2017, pp. 2106 & 2107)	No definition established. However, their results suggested that, “for some [men], sexual pleasure was about bonding, intimacy and closeness, while for others, the corporeal, embodied pleasures of orgasm were most central. […] ‘Pleasurable’ sex occurred for many of our participants at the nexus of intimacy, bonding and physical pleasure.”
Kelly et al. (2017, p. 249)	“Women might experience a form of ‘connection pleasure’ which we understood as the pleasure or satisfaction they derived from feeling connected and close to their partner while experiencing emotional intimacy.”
World Association of Sexual Health (WAS, 2019)	Definition adapted from GAB-definition, including following addition: “fantasies, emotions and feelings can also be sources of sexual pleasure.” (“Sexual pleasure is the physical and/or psychological satisfaction and enjoyment derived from shared or solitary erotic experiences, including thoughts, *fantasies*, dreams, *emotions, and feelings.”)* [italics added to highlight changes]
Halwani (2020, p. 122)	“Sexual pleasure (a) is usually both pleasure-as-sensation and pleasure-as-enjoyment, with (b) the former playing a central role in that the latter typically depends on it and in that it explains why people seek or avoid sexual activity; (c) sexperience machines and other far-fetched cases notwithstanding, it is not an entity detachable from the activity but supervenes on it; and (d) is generally distinguished from nonsexual pleasure through the notion of ‘arousal felt in the genitalia’, itself to be supplemented by a phenomenological or physiological account of ‘arousal’.”
Vigil et al. (2021, p. 327)	“[…] we operationalized sexual pleasure as conscious, positive evaluations of physical sensations during sex, either localized in the genitals or throughout the body.”

*Note.* We included statements which followed the structure of “Sexual pleasure is…”, or something similar, followed by a conceptual or explanatory definition, or themes which specify how sexual pleasure is defined by interviewees in qualitative studies. We have reviewed more articles than those presented in the table but excluded articles from the table which did not formulate a definition as specified above and articles which referred to one of the definitions in the table. For a list of the reviewed articles, see the supplementary material.

We noted two areas of convergence and divergence across the existing definitions: (1) sexual pleasure is conceptualized as unifaceted or multifaceted, and (2) can be conceptualized as a state or a trait.

(1) On the one hand, sexual pleasure is narrowly defined as a unifaceted, sensory or sensual, experience such as in the experience of pleasurable, enjoyable, or satisfactory “sex” and “sensations during sex”, or equated with the experience of orgasm. On the other hand, sexual pleasure is broadly defined as covering different types of experience such as cognitive and emotional experiences next to physical and sensory experiences. Theorists suggest that these different experiences result from and are related to different kinds of activities and sources (e.g., internal and external stimuli or stimulus situations, such as fantasy, tactile stimulation, physical closeness, intimacy, connection, bonding, safety, the partner’s pleasure, or spontaneity and flow). In other words, these conceptualizations suggest that there is either one kind or source of, or different kinds or sources of, sexual pleasure.

(2) Furthermore, some theorists have conceptualized sexual pleasure as an experience, or equivalent to a state of satisfaction or wellbeing derived from sexual activity, while others propose that, conceptually, sexual pleasure should also include a more trait-like psychological tendency or capacity (e.g., the ability to enjoy sex, or entitlement to and self-efficacy to enjoy). In other words, theorists suggest that sexual pleasure can be conceptualized as a momentary or contextual experience (a state) and a tendency for experiences (a trait). Thus, sexual pleasure has been defined as a *unifaceted* or *multifaceted state* as well as a *unifaceted* or *multifaceted trait*.

### Sexual pleasure: Proposed conceptual definitions

We propose that sexual pleasure should be conceptually, and especially operationally, defined as a multifaceted concept, encompassing several *state-like* and *trait-like* domains. At its core, we define state sexual pleasure as the *experience of positive affect (“feeling good”) during sexual activities* (a *positively valenced* emotional state; cf. Smuts, 2011). We argue that such positive affect is experienced when an activity is *rewarding*, that is, sexual pleasure is experienced when *anticipating and receiving rewards during sexual activities*. Such rewards are diverse, allowing for the multifaceted nature of state pleasure (see the section “A Multifaceted Taxonomy of Sexual Pleasure – Rewards Retrievable from Sexual Activity”). We define trait-like sexual pleasure as the *tendency to enjoy sexual activities,* that is, the tendency to experience state pleasure during sexual activities. This tendency is a function of the *contextual likelihood* to encounter rewarding sexual activities and the *capacity to enjoy* sexual activities. The capacity to enjoy sexual activities includes the (a) *propensities (“congenital predisposition”)* and (b) *abilities (“nurtured disposition”) to experience rewards* and the (c) *capabilities (“skills”) to attain the rewards* provided by sexual activities. We define sexual activity as all human actions which are geared toward or associated with *non/conscious central representations of genital arousal* within a *stimulus context which affords sexual construction of interoceptive experience*.

In sum, we propose that a person who (1) is (a) predisposed and learning to be (b) able and (c) capable of experiencing and attaining rewards during activities (2) which are associated with non/conscious central representations of genital arousal within stimulus contexts which afford sexual construction of interoceptive experience, and who (3) is given the opportunity to engage in such activities which also offer the conditions to experience and attain rewards (4) will experience sexual pleasure during these sexual activities, as long as inhibitory mechanisms are relatively less active.

### A note on state and trait sexual pleasure

Within both the available and our proposed definitions, we see that theorists have conceptualized sexual pleasure as a momentary experience or a tendency for experience. This conceptual difference has been referred to as state and trait conceptualizations of affective responses and has been particularly influential in operational definitions of affective responses. For instance, Spielberger (1972, 1983) conceptually and operationally differentiated state anxiety in response to a specific situation and trait proneness to experience anxiety in response to situations. Dawson and Chivers (2014) discussed state sexual desire in response to sexual stimulation and typical trait tendencies to experience sexual desire across situations. Differentiating between state and trait conceptualizations and operationalizations of sexual desire has led to crucial insights regarding the alleged difference in sexual desire between cis men and cis women. On average, cis women do not appear to differ from cis men in (momentary assessments of the level of) state sexual desire but do differ in (self-report assessments of) trait sexual desire (Dawson & Chivers, 2014; Frankenbach et al., 2022). Different definitions of a concept lead to different research conclusions about the concept.

Following our above definition, state sexual pleasure should, strictly, be conceptually defined as the experience of positive affect during *a concrete situation* in which sexual activity takes place *at a specific moment in time* (Dawson & Chivers, 2014; Schmitt & Blum, 2020). Trait sexual pleasure should be conceptually defined as the tendency to experience such state sexual pleasure across situations (Frankenbach et al., 2022). However, a sexual pleasure “trait” concept comprises two ways of conceptualizing the “trait” which results in “trait” sexual pleasure remaining ambiguous.

Traits can be *loosely* conceptualized as “dimensions of […] *relatively stable* psychological (affective, cognitive, motivational, and behavioral) *differences* among people” (Condon et al., 2020, p. 924, italicization added for clarification; Fleeson, 2001; Fleeson & Jayawickreme, 2015). A trait can also *strictly* be conceptualized as a “relatively stable, consistent, and enduring *internal characteristic* that is *inferred* from a pattern of behaviors, attitudes, feelings, and habits in the individual” (APA, n.d.-a) which “*[…] determines* an individual’s behavior across a range of situations” (APA, n.d.-b; italicization added for clarification). That is, a trait can be defined as a *summary* description of the typical experience of a person as well as an endogenous *causal* determinant of the experience of a person which is *inferred* from the pattern of the typical experience of a person (DeYoung, 2015; Fleeson, 2001; Fleeson & Jayawickreme, 2015).

In our proposed conceptual framework, we include both conceptualizations of traits: “loose” trait sexual pleasure as *individual differences in usually experienced state sexual pleasure across situations* and “strict” sexual pleasure traits as *individual differences in capacities to enjoy sexual activity*. Individual differences in usually experienced state sexual pleasure are a function of individual differences in the capacities to enjoy sexual activity *and* differences in the likelihood to encounter rewarding sexual situations. That is, individual differences in traits in the loose sense (the tendency to experience state sexual pleasure) do not only result from individual differences in the strict sense (the capacities to experience state sexual pleasure) but also systematic differences in the kind of situations people are (cap)able *and* allowed to encounter.

### State sexual pleasure as the affect component of sexual desire and lust

Several early theories of the sexual response and behavior, i.e., state sexual responses, can be understood from drive (reduction or induction) perspectives of motivation (APA, n.d.-c, n.d.-d) in which state sexual pleasure was mentioned only indirectly. Such perspectives assumed that sexual behavior is triggered when an organism’s internal equilibrium is disturbed, that sexual behavior aims to restore an organism to a sexual “set-point”, and that restoring such equilibrium is what is pleasurable about sexual experiences. For instance, Freud (1909) conceptualized sexual motivation as an internal “continuously increasing” force, a state of “libidinous” tension, which can be relieved through “unburdening of the seminal vesicles” (pp. 148-149; see also, Both et al., 2005; Everaerd et al., 2001). Notably, Masters and Johnson’s sexual response curve (Masters & Johnson, 1966) and its adaptations by Kaplan (1979) and Levin (2001) seemed to imply that release or reduction of arousal/tension equals pleasure, embodied within the experience of orgasm. Even though these perspectives differed in terms of how the arousal/tension was set in motion – either being triggered by deprivation or by an arousing stimulus – they all implied that pleasure was a side-effect of quenching internal arousal/tension (see also Janssen, 2011, and the historical overview in Toates, 2014, and Pfaus, 1999; as well as Hilgard & Marquis, 1961a; Hilgard & Marquis, 1961b). These theories thereby implied that all kinds of arousal/tension release are equally pleasurable and that pleasure only figures at the end of the sexual response. As an unfortunate yet crucial consequence, these theories of sexual responding hid pleasure from conceptualization, because pleasure hides within the unified construct of diminishing arousal (Janssen, 2011).

Incentive Motivation Theory (IMT) of sexual response and behavior combined and furthered aspects of these perspectives (Berridge, 2018; Bindra, 1978; Singer & Toates, 1987) which set the stage for a broader perspective on pleasure to step into conceptual focus. IMT proposed that sexual responding is not only dependent on characteristics of the organism (e.g., its’ deprivation) as drive reduction theories suggested, nor that it is only dependent on (un)conditioned stimulus characteristics inducing responses and reactions as drive induction theories suggested (i.e., stimulus characteristics). According to IMT, an organism learns when to predict and expect, and when and how to attain and consume stimuli that induce *reward.* However, it depends on the current sensitivity of the organism whether stimuli are being processed as rewarding and on the availability of rewarding stimulus situations in the environment (Berridge, 2018, 2019; Bindra, 1978; Both et al., 2005; Laan & Both, 2008; Singer & Toates, 1987; Toates, 2009, 2014).

Initially, IMT and its predecessors did not clearly specify why a rewarding stimulus is experienced as “rewarding” (Bindra, 1978; Hilgard & Marquis, 1961a; 1961b; Singer & Toates, 1987). In 1993, Robinson and Berridge’s reward-behavior cycle specified the consequences of interaction with unconditioned and conditioned rewarding stimuli into *wanting* and *liking* responses during the anticipation, attainment, and consumption of such “rewarding”, i.e., wanting and liking inducing, stimuli. *Wanting* is related to the (previously experienced) salience of the reward and reflected in the action readiness and sustenance exerted in response to signals of reward and punishment (Berridge, 1996, 2019; Gola et al., 2016). *Liking* is related to the (previously experienced) positive valence of the rewarding stimulus and reflected in the hedonic impact of anticipating,^1^ attaining, and consuming a reward, and is considered synonymous with non/conscious pleasure (Berridge & Kringelbach, 2009; Georgiadis et al., 2012; Georgiadis & Kringelbach, 2012). That is, a reward compels action and is experienced as positive.

IMT, in combination with wanting and liking from the reward-behavior cycle, allows us to conceptualize the sexual response as an “emo(tiva)tional” affective state that emerges when an individual experiences changes in action preparation, action readiness, and action evaluation (Frijda, 1988, p. 493; cited in Henckens & Everaerd, 2020; Henckens et al., 2020). That is, the sexual response can be conceptualized as an affective response (cf. Barrett & Simmons, 2015; Berridge, 2018; Frijda, 1993; LeDoux, 2012), which *emerges* (Barrett, 2013, 2009; Singer & Toates, 1987) from arousal^2^ *and* anticipatory and consummatory reward (wanting + liking) derived by the organism during sexual stimulus processing (Everaerd, 2002; Frijda, 1993; Henckens & Everaerd, 2020; Janssen et al., 2000; Smid & Wever, 2019). When arousal and anticipatory reward processes during stimulus processing reach consciousness, the organism may experience the nonconscious emotivation as the consciously emergent feeling of desire (cf. Everaerd, 2003, p. 85; Hermans et al., 2013, cited in Henckens & Everaerd, 2020).

We therefore suggest that a “sexual” stimulus (situation) could be characterized by three overarching aspects: (1) its “sensory intensity” (defined by changes in arousal), (2) its reward “competence” which includes (a) its salience (defined by changes in wanting) and (b) (positive) valence (defined by changes in liking) and (3) its “relevance” (defined by changes in sexual meaning/connotation). Sensory stimulus “intensity” is associated with changes in the strength of (central nervous system representation of) physiological, including genital, *arousal* (cf. Ågmo, 2008, 2011; De la Garza-Mercer, 2007; Halwani, 2020; Hoffmann, 2017; Paredes & Ågmo, 2004; Pfaff, 1999; Toates, 2009). A stimulus is or becomes “competent” (Damasio, 2001) when the stimulus signals the availability of or represents a conditioned or unconditioned reward (cf. Both et al., 2020; Oei et al., 2014). Stimuli are and become “relevant” by socioculturally reinforced sexual meanings/connotations during sexual development (cf. Barrett, 2013; Gripsrud, 2008; Jackson & Scott, 2007; Toates, 2014).

Practically, these conceptual distinctions allow us to hypothesize how the sexual response might be(come) “dysfunctional”. For instance, when someone perceives physiological, including genital, arousal – a phenomenon which is sometimes called *subjective* arousal (cf. Meston & Stanton, 2019) – they do not necessarily experience desire, since they might not associate sufficient reward value with the stimulus triggering the arousal; nor would they experience desire when experiencing wanting without sufficient anticipatory liking, because it might be experienced as urge rather than desire (cf. Briken, 2020; Prause et al., 2017). Also, cis women and cis men might differ in concordance, i.e., their reporting of subjective arousal to visual stimuli even though they show a comparable genital response, because what they report is the difference between arousal versus arousal + reward (wanting and liking) with women potentially responding with less reward to (non-self-selected, i.e., potentially incompetent) visual sexual stimuli (cf. Maunder et al., 2022).

**Figure 1.**
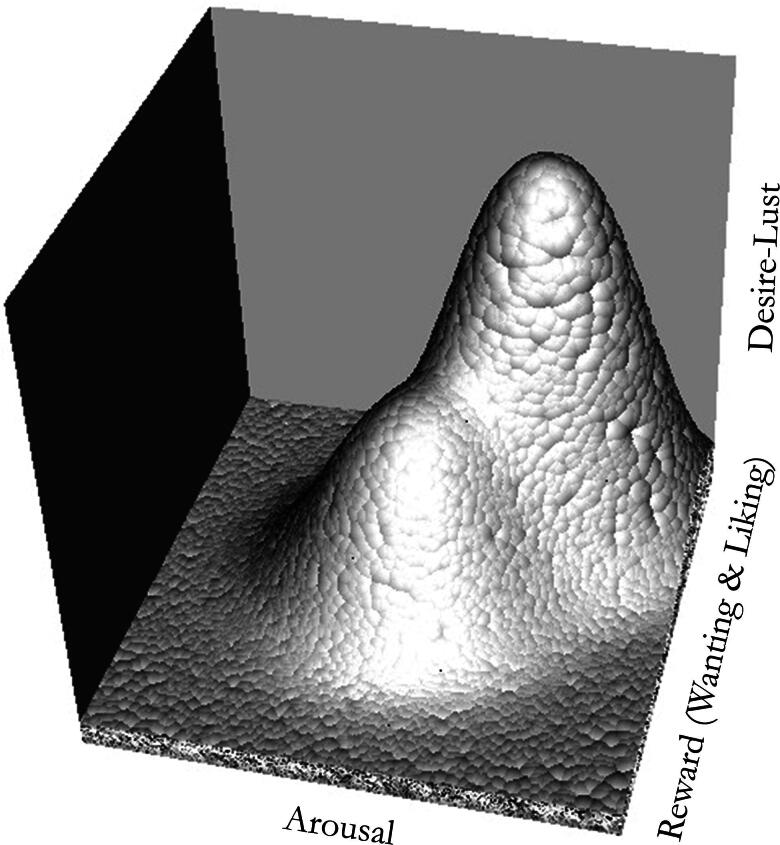
A Sexual Response Surface. *Note.* This graphic represents an illustrative visualization of a sexual response surface. This representation should not be overinterpreted as its shape needs to be verified by simulations and modeling of empirical data. For instance, a cusp surface (Huby, 1991; Levin, 2017) might better represent what happens during sexual responding, rather than the shape which is visualized here for illustrative purposes.

In sum, pleasure is not a mere side-effect of arousal/tension reduction. Pleasure, i.e., anticipatory and consummatory liking, is experienced when anticipating, attaining, and consuming rewarding stimuli. For sexual desire to emerge,^3^ a sensitive organism has to be triggered by arousing and rewarding sexual stimuli, which have attained or (de)potentiated their intensity, competence, and relevance through their developmental conditioning history. Desire does not emerge from increasing tension, but in response to the expectation of rewarding sex (Halwani, 2020). Thus, synthesizing sexual response curves à la Masters and Johnson with the reward-behavior-based sexual pleasure cycle (Georgiadis et al., 2012; Georgiadis & Kringelbach, 2012; Robinson & Berridge, 1993), we suggest that the sexual response should be visualized as a surface rather than curve (see Figure 1), encompassing stimulus-induced arousal *and* reward, emerging into anticipatory desire and consummatory lust (see also the discussion of “erotic sensuality” in Komisaruk & Rodriguez del Cerro, 2021).

### Sexual responsiveness – Trait sensitivity to arousing or rewarding stimuli, or both?

To experience a sexual response, IMT argues that an organism needs to be sufficiently responsive to detect and respond to stimulus characteristics. IMT proposes that the sexual response system’s “sensitivity of incentive motivational circuitry” (Toates, 2009, p. 175) determines the sexual system’s responsiveness to sexual stimuli and thereby shapes its sexual response output. The inclusion of such an individual-difference concept connects the *state* of sexual responding (experience of arousal, wanting, and liking) within an intense and competent sexual context to the strict *traits* of the sexual response system (differences in responsiveness due to differences in sensitivity; cf. also Byrne & Schultz, 1990; Cervone, 2004; both cited in Gijs et al., 2009). Thus, there are not only intra-individual and inter-individual differences in the sexual response due to differences in the availability and strength of intense and competent sexual stimuli, but also trait-like intra-individual and inter-individual differences that relate to differences in sexual responsiveness to the same stimulus situation (within people over time and between people at a time, respectively).

A frequently utilized and researched strict trait-like concept, similar to sexual *responsiveness*, is called sexual *excitation*. According to the Dual Control Model of Sexual Response (Janssen & Bancroft, 2007), sexual excitation and sexual inhibition represent “two neurophysiological systems, one relevant to activation and the other to suppression of sexual response” (p. 199). However, the “sensitivity of incentive motivational circuitry” (Toates, 2009, p. 175) seems to denote a different trait-like concept than sexual excitation. While the “sensitivity of incentive motivational circuitry” seems to connote *reward*, and specifically *wanting* sensitivity (Toates, 2009, p. 173; 2014, p. 143), sexual excitation seems to connote sexual *arousa*bility (cf. Whalen, 1966) with sexual arousability either denoting *arousal* and/or *wanting* sensitivity. Most importantly, intra-individual and inter-individual differences in *liking* (i.e., pleasure) hide from view yet again because the theories focus on intra-individual and inter-individual differences in arousal and wanting, but not liking, sensitivity. Such conceptual and verbal conflation might result from the previously mentioned fact that the sexual response, à la Masters and Johnson, has traditionally been seen to encompass only one, potentially all-encompassing, output – sexual arousal – rather than arousal *and* reward (wanting and liking).

**Figure 2.**
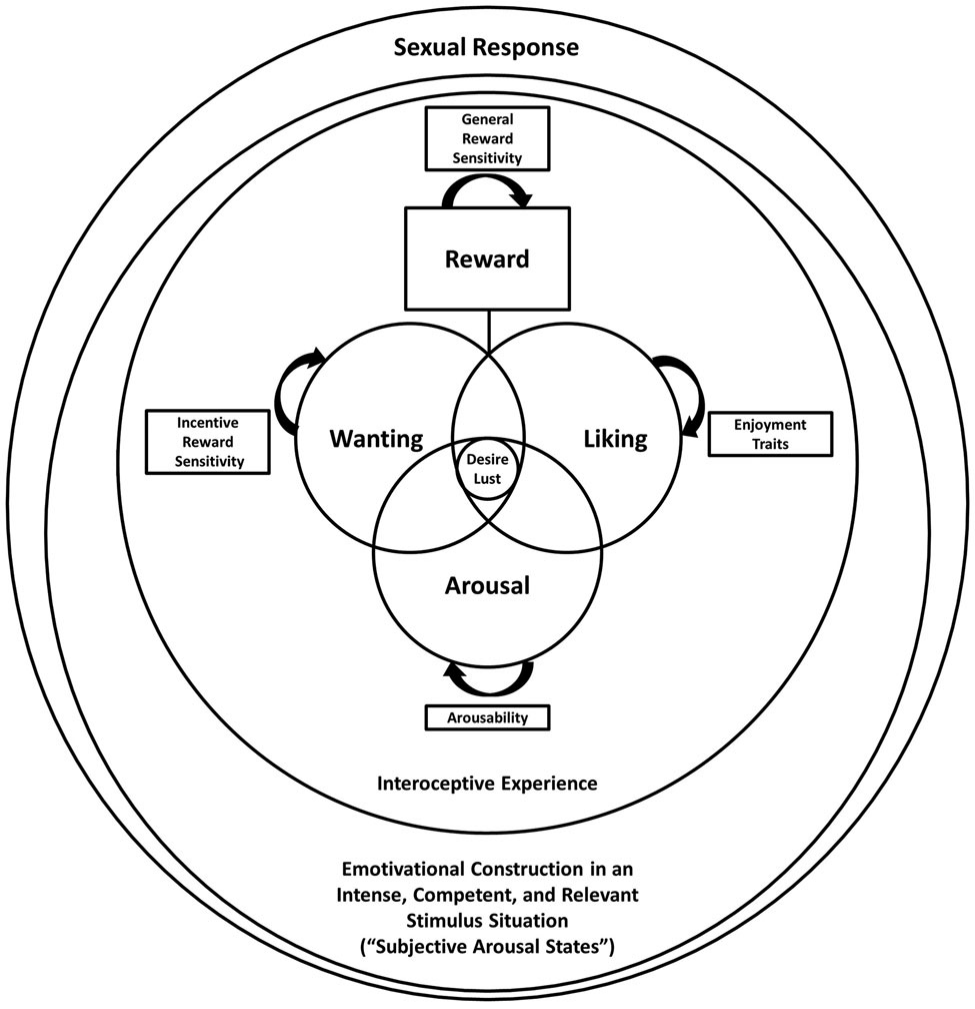
A Diagram of Sexual Response Concepts. *Note.* The diagram does not specify causal mechanisms but conceptual relations and hierarchies.

We are not the first to (re)iterate a difference, at least conceptually, between arousal and reward circuitries, and that each of these could exhibit strict trait-like differences (Carver & White, 1994; Corr, 2009; Eysenck, 1967; Frijda, 2008; Gray, 1982; Henckens & Everaerd, 2020; Janssen & Bancroft, 2007; Whalen, 1966). For instance, we might call the overall state-output of the whole sexual response system its sexual *response* and its individual trait-difference sexual *responsiveness*. Sexual excitation in interaction with sexual inhibition (Janssen & Bancroft, 2007) might validly denote individual differences in overall responsiveness, since their interaction seems to encompass individual differences across all state circuitries, i.e., arousal, wanting, and liking, as well as aversion-circuitries (Bancroft, 1999; sometimes referred to as sensitivity of the overall nervous system; Toates, 2009, p. 170). We would suggest that arousability (Eysenck, 1967; Janssen & Bancroft, 2007; Whalen, 1966) denotes trait-like differences of the arousal circuitry, that incentive reward sensitivity (DeYoung, 2015) denotes trait-like differences in wanting circuitry, and that general reward sensitivity (Toates, 2009) denotes trait-like differences across both wanting and liking circuitries. We will discuss our suggestion for trait-like differences in liking in the following sections.^4^ Figure 2 offers a visual overview of our conceptual suggestions.

### Learning when and where to attain rewarding sex

According to IMT, the sensitivity to incentives and rewards is determined by nutrient deficits and hormonal sensitization (Berridge, 2001; Bindra, 1978; Toates, 1986). This implies that the (strict trait) sensitivity, rather than being fixed across time, can change according to nutrient deficits and changes in hormonal milieu. Importantly, Both et al. (2007, p. 329) argued that “sexual motivation does not emerge through a [nutrient] deficit signaled by the hypothalamus” since there is no sexual nutrient deficit or sexual tissue need which would require the hypothalamus to signal a survival emergency when such “sexual set-points” deviate from some needed level (see also, Hardy, 1964, cited in Hardy, 1989; Beach, 1956, cited in Singer & Toates, 1987). It follows that the sensitivity of the sexual response system changes through hormonal sensitization but that this hormonal sensitization adapts by another regulatory mechanism than hypothalamic homeostasis (Berntson & Cacioppo, 2007).

We suggest that the sexual response system regulates arousal, wanting, and liking states homeostatically, i.e., it reacts and corrects, *but* that it adapts, i.e., anticipates, prepares, and adjusts, its trait sensitivities heterostatically (or allostatically, as some might want to call it; see Quintana & Guastella, 2020, for the inspiration to this argument; as well as Schulkin, 2003, and Caldwell, 2002) through learning mechanisms. IMT implies that organisms do not only sexually react once an unconditioned stimulus is present, but that they anticipate and prepare metabolically and energetically to the expectation of unconditioned positive and negative *future* deviations in the environment (i.e., opportunities and threats) based on *previous* sexual experiences (Barrett & Simmons, 2015; Schulkin, 2003). We suggest that the capacity to anticipate and prepare for a rewarding opportunity, i.e., respond with wanting and liking to the anticipation of rewards, is enabled by a sensitized sexual response system which adapts its sensitivities according to experience with rewarding sexual experiences in certain environments (Ramsay & Woods, 2016).

As a result, the sexual response can be conceptualized as circular across time (Basson, 2000; Hayes, 2011) rather than linear (Kaplan, 1979; Levin, 2001; Masters & Johnson, 1966), because sexual learning attaches feed-forward loops to the sexual response (Ågmo & Laan, 2022), with the experiences during a sexual response event affecting sexual response events in the future through, among others, adaptation of the sensitivities of the sexual response system (Ramsay & Woods, 2016). Similarly, Basson (2000) visualized the female sexual response as a cycle with stimuli and experiences playing a role in feed-forward processes, in contrast to the sequential, linear, and self-contained process implied by sexual response curves. Relatedly, we argue that learning from sexual events changes future sexual stimulus aspects through classical and operant associative conditioning, *and* through adaptation of the traits of the sexual response system, such as its reward sensitivity (Ramsay & Woods, 2016; cf. Meston, 2000; Henckens & Everaerd, 2020; Goldey & van Anders, 2015; Macoveanu et al., 2016; Tobiansky et al., 2018). Contrary to Basson (2000), we would suggest that such feed-forward adaptation is applicable to all (rather than only female) sexual response systems.

In sum, humans do not need to have sex periodically to survive, but *become* sensitive to signals in the environment that propose a potentially valuable opportunity for procreation and recreation and *learn* that such opportunities are available and how to attain them. Learning changes the competence of stimuli by associative learning, by teaching an individual how and where to attain rewards, and by sensitizing an individual’s response system in an environment rich with rewarding opportunities.

## Aim 2: A multifaceted taxonomy of sexual pleasure – Rewards retrievable from sexual activity

### Pleasure as state – The experience of liking in response to rewards

State liking and wanting specify what processes make a reward rewarding, and learning and conditioning principles describe how stimuli can take on reward value and become incentives. However, how do rewards become rewarding? What is the unconditioned stimulus that triggers state liking and wanting without any previous learning history such that unconditioned liking and wanting can be conferred from the unconditioned stimulus to conditioned stimuli (Hilgard & Marquis, 1961a; 1961b)? In other words, are there unconditioned, i.e., primary, or at least universal rewards experienced during sexual activities which all humans (and potentially, mammals) like and want during sexual activity?

Research in rodents suggests that liking associated with orgasm reflects a critical component of reward and reinforcement, which represents strong evidence for orgasm being an unconditioned reward. (Pleasurable) Orgasm is associated with strong endogenous opioid release and opioid action appears necessary for learning through sexual experience (Ågmo & Berenfeld, 1990; Georgiadis & Kringelbach, 2012; Paredes, 2014; Pfaus et al., 2012). However, we suggest that state sexual pleasure encompasses *any experience of state liking during sexual activity*, i.e., not only the anticipatory and consummatory liking connected to or triggered by anticipation of the potentially primary rewarding stimulus situation of orgasm. As Goldey et al. (2016) and Fileborn et al. (2017) pointed out, orgasm represents only one of multiple experienced rewards during sex (see also the discussion in *Sexual Pleasure versus Pleasure during Sex* below and critiques by Tiefer, 2004, and Fahs & Plante, 2017, Opperman et al., 2014, and Kleinplatz et al., 2009). We therefore suggest that, since liking is experienced when anticipating, attaining, and consuming rewards, *any reward* anticipation, attainment, or consumption *during sex* should be able to trigger *pleasure during sex* (cf. Smuts, 2011). The question then becomes what kinds of rewards, next to orgasm, humans anticipate, attain, and consume during or via sexual activity.

To identify these potential rewards, we refer to the literature we label the *basic sexual and psychological rewards literature* (which is usually called the basic *needs* literature; Prentice et al., 2014).^5^ In the “sexual” rewards literature, Van Anders et al. (2011) noted that sex is rewarding because it offers erotic and nurturance rewards (see also, Diamond, 2003; Goldey et al., 2016; Toates, 2009). Following their Steroid/Peptide Theory, they argue that erotic rewards are (evolved to be) rewarding to facilitate reproduction, while nurturance rewards are (evolved to be) rewarding to facilitate parent-offspring and couple attachment (Diamond, 2003; Van Anders, 2015; Van Anders et al., 2011). In addition, Goldey et al.’s (2016) interviewees noted that pleasuring and sharing pleasure with the partner was experienced as rewarding, as well as feeling autonomous and explorative during sex (see Hargons et al., 2018 and Pascoal et al., 2014, for similar findings, and Opperman et al., 2014 and Brown et al., 2018, for the importance of shared pleasure). In an exploratory study, Werner et al. (in preparation) factor-analyzed and summarized a broad list of items referring to different rewarding aspects of sexual activity into domains referring to pleasure retrieved from arousal, pleasure retrieved from being intimate and connecting with sexual partners, pleasure retrieved from pleasuring sexual partners, and feeling competent and confident about oneself and one’s body. Additional studies referred to in Table 1 pointed to feeling connected and experiencing ease and flow as rewarding aspects of sexual activity.

In the “psychological” rewards literature, such as the framework of Self-Determination Theory (Ryan & Deci, 2000), autonomy, competence, and relatedness are said to be rewarding, suggesting that feeling un-coerced and in control during sex (autonomy), engaging sexual skills (competence), and connecting and cooperating with sexual partners (relatedness; Smith, 2007) act as rewarding aspects of sexual activity. According to Maslow (1943) there are basic physiological, safety, love, esteem, and self-actualization rewards. According to Grawe (2004) pleasure, attachment, self-esteem enhancement, and orientation & control are rewarding. Physiological rewards and pleasure refer to the fact that being in physiologically pleasurable states is rewarding; safety refers to reward experienced from being in a protective and predictable environment; love and attachment refer to the rewarding state of building and experiencing a positive relationship with a reference person; self-esteem enhancement proposes that having and building a positive self-image is rewarding; orientation and control refers to the rewarding state of feeling able to control one’s own environment by taking action (Peters & Ghadiri, 2013); and self-actualization refers to the reward experienced from authentic peak experiences (APA, n.d.-e). Dweck (2017) suggested acceptance, predictability, and competence leading to control, self-esteem and trust as basic rewards. Talevich et al. (2017) suggested meaning, communion, and agency as basic rewards. A recent special issue edited by Vansteenkiste et al. (2020) argued that beneficence (“individual’s perception of having a positive impact on others”), novelty (“individual’s perception of experiencing or doing something new”), and morality (“individual’s perception of being and acting morally”) could be added to the basic reward list.

We integrate all of these “sexual” and “basic psychological” rewards into the following taxonomy of rewards that might be anticipated, attained, and consumed during sexual activities and thereby induce the experience of state pleasure during sex (see also Table 2 and Habermacher et al., 2014, 2020; and Pittman & Zeigler, 2007 for a similar synthesis in a nonsexual domain): *Sensual Pleasure* (encompassing basic sensory, physiological as well as erotic rewards), *Bonding Pleasure* (encompassing nurturance, relatedness, connection, love, acceptance, communion, and attachment and parts of trust and safety), *Interaction Pleasure* (encompassing sharing pleasure, relatedness, beneficence, and parts of morality), *Pleasure-related Validation* (encompassing esteem and self-esteem enhancement) and *Pleasure-related Mastery* (encompassing competence, orientation & control, agency, and parts of autonomy, predictability, control, and self-actualization). That is, we conclude that sex serves, and can be coopted to serve, sensual rewards, bonding rewards, interaction rewards, and self-validation and mastery.

**Table 2.** Proposed Domains of (the Loose Trait Tendency for) State Sexual Pleasure and (Strict) Sexual Pleasure Traits.

Domain	(Strict/Loose) Trait facets	State facets
Hedonic Domain	*Arousal Enjoyment* The ability/tendency to enjoy sensual stimulation and its psychophysiological consequences.	*Sensual Pleasure* Level of experienced pleasure through sensual stimulation and its psychophysiological consequences.
Interpersonal Domain	*Bonding Enjoyment* The ability/tendency to experience and enjoy the bonding-related rewards of sexual interactions.	*Bonding Pleasure* Level of experienced (pleasure through) feelings of closeness, affection, safety, and security during sexual interactions.
	*Interaction Enjoyment* The capability/tendency to enjoy pleasuring and being pleasured by a sexual partner (i.e., enjoying the sharing of pleasure).	*Interaction Pleasure* Level of pleasure experienced during sharing pleasure and from interaction with a sexual partner.
Intrapersonal Domain	*Enjoyment-related Self-Efficacy* Self-perceived confidence and competence (knowledge and skills on how) to engage in pleasurable sexual activities./ The tendency to be confident and competent about engaging in pleasurable sexual activities.	*Pleasure-related Mastery* Level of experienced mastery in creating pleasurable sexual activities.
	*Enjoyment-related Self-Worth* Evaluation of one’s sexual worthiness and feeling deserving of positive sexual experiences. /The tendency to evaluate oneself as sexually worthy and deserving of positive sexual experiences.	*Pleasure-related Validation* Level of perceived worthiness to experience positive sexual experiences and experienced self-validation during sex.

*Note.* Note that the state-domains include the word pleasure, while the trait-domains include the word enjoyment and that we differentiate between abilities (as more trait-like dispositions for experience) and capabilities (as more trait-like skills to bring about experience). Hereby, we aim to stress the difference between states of experience versus strict traits for bringing about experience.

Note that probably not all of these rewards need to be experienced to their full extent during sexual activity to experience pleasure during sex. It is likely that the state pleasure facets do not act as summative components that can be added up to express ever more satisfying or healthy forms of sexual experience. Future research needs to establish when, to what extent, and in what constellation the rewards result in “good-enough” (Metz, & McCarthy, 2007) or “optimal” sex (Kleinplatz et al., 2009). For instance, some people might use sex more or less to retrieve bonding-related rewards, and some might do so only in certain situations or relationships. Specifically, we would suggest that sexual preferences (e.g., Hill, 2021), general personality traits (Barlow, 1986; Nobre, 2013), and “key partner/context/event-specific enabling factors” (Fava & Fortenberry, 2021; GAB, 2016) interact with the experience of rewards to predict sexual satisfaction, health, and wellbeing. We have not (yet) fully included autonomy and predictability, ease and flow, exploration and novelty, and self-actualization and meaning in the taxonomy. We discuss the reasons for these decisions in the discussion.

### Strict pleasure traits – The capacity to experience liking (attain and experience rewards) during sex

We discussed that strict traits of the organism’s sexual system explain why there are differences in the sexual response between people at a moment in time and within people over time in response to equally competent stimulus situations. We also argued that just because such traits might be prepared and more stable than state-like experiences, this does not imply that such traits do not adapt to experience. We argue that learning from experience modifies the capacities of the organism, which includes the sensitivity to rewards, in addition to other pleasure-related abilities and capabilities of the individual. Thus, we suggest that the state experience of pleasure (through the anticipation, attainment, and consumption of sensual, bonding, and interaction rewards and mastery and validation) is a function of such rewards being available during sexual activities (the situation) and certain prepared and adapted trait-like capacities of the individual (the person), just like the experience of response is dependent on the availability of an intense, competent, and relevant stimulus situation (the situation) and the current responsiveness of the organism (the person) which make them (cap)able to attain and experience arousal and reward.

For someone to respond with the experience of sensual pleasure to an intense, competent, and relevant stimulus (situation), we suggest that the organism’s arousal circuitry would have to be sufficiently arousable and the reward system would have to be sufficiently sensitized. The latter implies that the individual would need to have experienced certain kinds of stimulation as something that brings reward or would need a reward system that is (consistently) sensitized by other means (for instance, drugs; Lorvick et al., 2012; menstrual cycle effects, or spillover from other environmental triggers; Toates, 2014). We label the ability to enjoy stimulation *Arousal Enjoyment* and define it as the ability to enjoy sensual stimulation and its psychophysiological consequences. We define sensual stimulation as exposure to external and internal stimuli of varying modalities/senses. In sum, an individual needs to exhibit the propensity to be sensually stimulated (have an arousable arousal system), and needs to have developed the ability to enjoy, i.e., respond with anticipatory and consummatory liking to, such stimulation.

Similar to sensual pleasure, we suggest that the experience of pleasure related to bonding depends on the human propensity to bond and a person’s developed attachment strategies. Attachment strategies are reflected within someone’s attachment style, which is based on positive experiences within bonds with caretakers (Dewitte, 2012, 2014; Hazan & Shaver, 1987, 1994). We label the ability to enjoy bonding during sex *Bonding Enjoyment* and define it as the ability to experience and enjoy the bonding-related rewards of sexual interactions. That is, the individual needs to have the propensity to attach and needs to have developed the ability to feel attached and enjoy bonding-related rewards during sexual activity.

We suggest that the tendency to feel *Pleasure-related Mastery* during sexual activity is facilitated by the capability we label *Enjoyment-related Self-Efficacy* (cf. Bandura, 2006; Horne & Zimmer-Gembeck, 2006) and that the tendency to feel *Pleasure-related Validation* is facilitated by the ability we label *Enjoyment-related Self-Worth* (cf. Horne & Zimmer-Gembeck, 2006). We define *Enjoyment-related Self-Efficacy* as the confidence and competence (knowledge and skills on how) to engage in pleasurable sexual activities and *Enjoyment-related Self-Worth* as the evaluation of one’s sexual worthiness and feeling deserving of positive sexual experiences. Finally, we label the capability which facilitates the experience of *Interaction Rewards* as *Interaction Enjoyment* and define it as the capability to enjoy pleasuring and being pleasured by a sexual partner, i.e., the capability to enjoy the sharing of pleasure (cf. Brown et al., 2018; Muise et al., 2013; Opperman et al., 2014). We would suggest that *Enjoyment-related Self-Efficacy* and *Enjoyment-related Self-Worth* also facilitate the experience of the other rewards, since knowing how to create a sexually competent situation and feeling deserving of such experiences should facilitate experiencing sexually competent situations.

**Figure 3.**
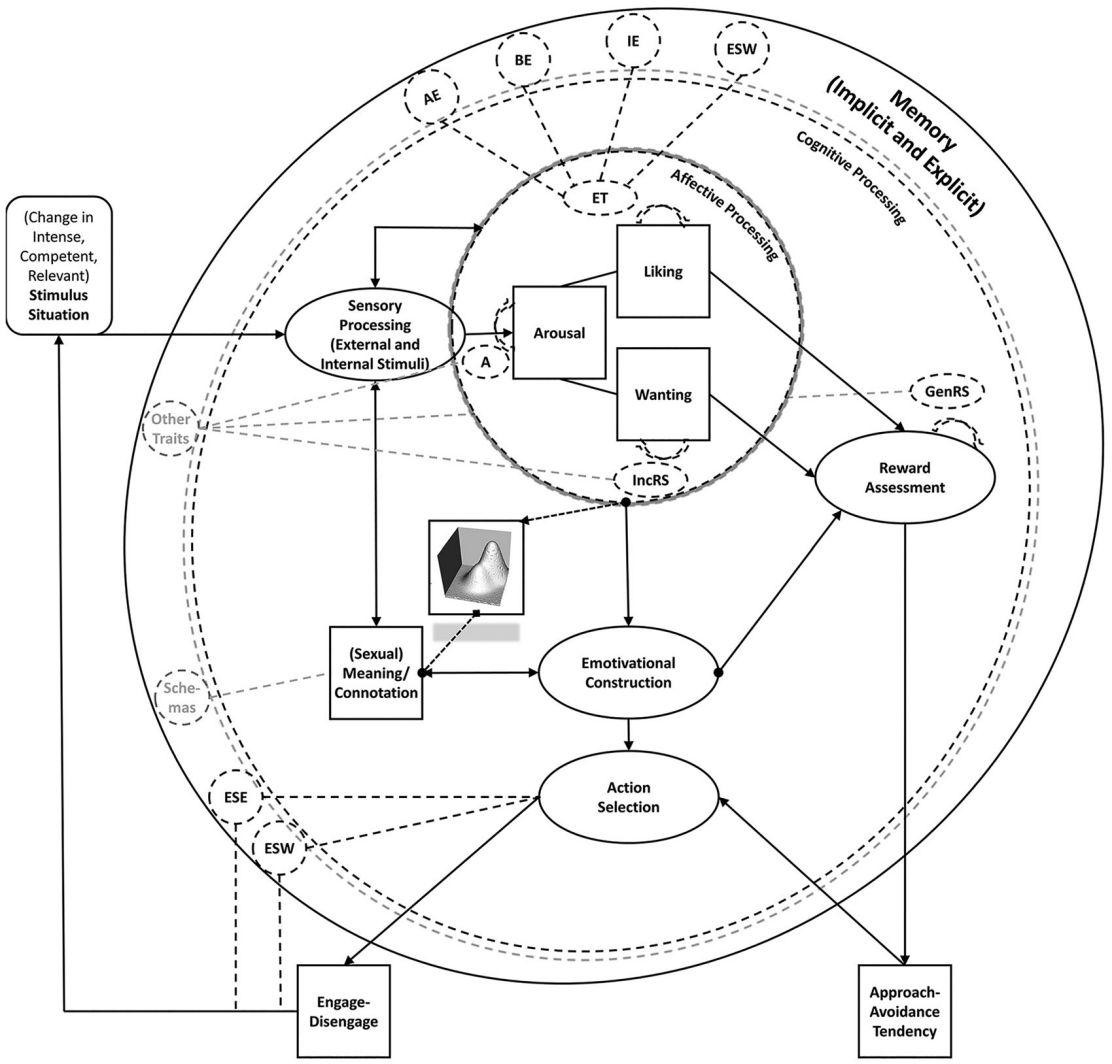
A (Work in Progress) Diagram of the Sexual Response System. *Note.* This diagram is based on and inspired by the work of Toates (2009), Bancroft (1999), Everaerd et al. (2001), Janssen et al. (2000), Barrett (2017), DeYoung (2015), and Robinaugh et al. (2019). AE = Arousal Enjoyment; BE = Bonding Enjoyment; IE = Interaction Enjoyment; ESW = Enjoyment-related Self-Worth; ESE = Enjoyment-related Self-Efficacy; ET = Enjoyment Traits; A = Arousability; IncRS = Incentive Reward Sensitivity; GenRS = General Reward Sensitivity. Double arrows indicate that the respective processes feed back into each other during processing. Strict traits act as parameters of the functioning of the state-processes (how strongly, quickly, frequently these re/act).^4^ The sexual response surface is added to show that the response emerges as the output of affective processing within context through emotivational construction. The diagram is not exhaustive. For instance, future diagrams need to incorporate inhibitory processes.

In Table 2 we provide an overview of all state and trait pleasure domains. Table 2 also aims to point out the subtle but crucial conceptual difference between the strict and loose traits, with strict traits referring to the above trait capacities for pleasure, while loose traits refer to the tendency to experience state pleasure associated with the different rewards across situations (see also Figure 5 in a later section). In Figure 3, we visually summarize a work-in-progress sexual response system in which we indicate where the strict sexual pleasure traits might potentially modulate state sexual responding.

### What sexual pleasure is (not)

#### Sexual pleasure versus pleasure during sex

Some readers might argue that what we come to call sexual pleasure is not sexual pleasure and that only sensual pleasure should be labeled sexual pleasure. This issue partakes in the discussion between essentialist views on basic emotions versus constructionist views of emotions (Gendron & Barrett, 2009; Stevenson et al., 2011). Across these schools of thought, we ask what essentially defines a *sexual* experience and what separates a *sexual* from *other* emotivational experiences. That is, we ask whether all emotivations have distinct and unique brain modules, physiological fingerprints, and activating stimuli that differentiate them from each other, or whether there are basic affective ingredients (e.g., arousal, wanting, liking) which emerge into an emotivational experience of fear, anger, or sexual desire based on embodied emotivational states within a context (see Barrett, 2017, 2006; Barrett et al., 2015; Berridge, 2018, 2019; Mosher & MacIan, 1994; Peterson & Janssen, 2007, cited in Stevenson et al., 2011).

The underlying question of such readers might be what the primary liking-inducing *sexual* reward is which then induces *sexual* pleasure rather than pleasure associated with enjoying food or scratching an itch (Komisaruk & Rodriguez del Cerro, 2021). We assume many would answer sensual rewards and sensual pleasure, including orgasm. However, using orgasm-related reward to define sexual pleasure assumes that orgasms and its prequels are inherently positively valenced and inherently sexually relevant (cf. Prause, 2011) – assumptions which can be countered with four phenomena. First, (reflex-)orgasms can be experienced during rape but are perceived as an aversive experience (see for a review Levin & van Berlo, 2004) potentially because general, including genital, arousal and potentially negatively valenced wanting (i.e., urge to fight, flee, freeze) are triggered and perceived, but without an association with liking (absence of positive valence; Sugrue & Whipple, 2001). Second, orgasm (incl. ejaculation) can occur without the experience of pleasure associated with this type of orgasm, also called anhedonic or pleasure dissociative orgasm (e.g., Parish et al., 2021; Rosenbaum & Pollack, 1988). Third, orgasms can be experienced as aversive even in consensual sexual encounters (Chadwick et al., 2019). Fourth, pleasurable orgasms can be experienced outside of sexually relevant situations and in absence of frequently used forms of stimulation (e.g., Austin, 2016; Kinsey et al., 1998; Wells, 1983). That is, orgasms are not necessarily pleasurable, and pleasurable orgasms are not necessarily sexual; liking during an experience constructed as sexual is. It then becomes a conceptual discussion whether sexual pleasure shall be reserved to liking that is related to (the anticipation of) the sensations of pleasurable orgasm or whether any liking induced by rewards *during* sex can be called sexual pleasure (cf. Boul et al., 2009), as long as the emotivational experience which emerges from such liking is constructed to be sexual (see also, Hoffmann, 2017, for an extensive review on the learning of response and action patterns that many might consider “primary” or “innate”).

Importantly, specifically sensual pleasure has been shown to associate with painless penetrative intercourse (Brauer et al., 2014) potentially due to opioid-related analgesic effects (Gianotten et al., 2021; Paredes, 2014) and enhanced genital arousal (Toates, 2009, p. 170 & 173). Thus, sensual pleasure during sex appears to be a prerequisite for those who want to practice penetrative sexual activity. However, integrating the above reminds us that it is not only about sensual rewards but also other types of rewards that induce liking during sex and that are retrieved through sexual activity, especially if we broaden sexual activity to encompass more than penetration. We argue that *liking* during sex is *liking* during sex, irrespective of which reward induces it, and that pleasure during sex can be induced by a variety of rewards. People (can) choose from a buffet of options, as long as they are allowed, able, and capable to cook with each other or for themselves.

#### Sexual pleasure, sexual satisfaction, sexual health, and sexual wellbeing

Furthermore, sexual pleasure is often conflated with sexual satisfaction because satisfaction can be defined as reward gratification (i.e., satiety trough “drive reduction”) which is understood as pleasurable. However, sexual pleasure differs from sexual satisfaction, because satisfaction seems to reflect someone’s evaluative balance-sheet between expectations and perceived reality rather than their actual experience of rewards (McClelland, 2010, 2011). For instance, someone who repeatedly experiences pain during sexual activities might be satisfied once such pain ceases, rather than that such satisfaction reflects that they experience rewards during sexual activities. Just as the absence of disease does not define health (WHO, 2002), so does the absence of pain or aversion not define pleasure.

Sexual pleasure should also be distinguished from sexual health and sexual function. Thanks to the WHO and WAS, sexual pleasure has been recognized as part of sexual health, which implies that sexual health encompasses more than sexual pleasure and that the terms should not be used synonymously (Fava & Fortenberry, 2021). Also, research has repeatedly shown that it is foremost a lack of pleasure associated with sexual dysfunction that predicts sexual distress, rather than the loss of sexual function per se (Pascoal et al., 2020; Stephenson & Meston, 2015).

**Figure 4.**
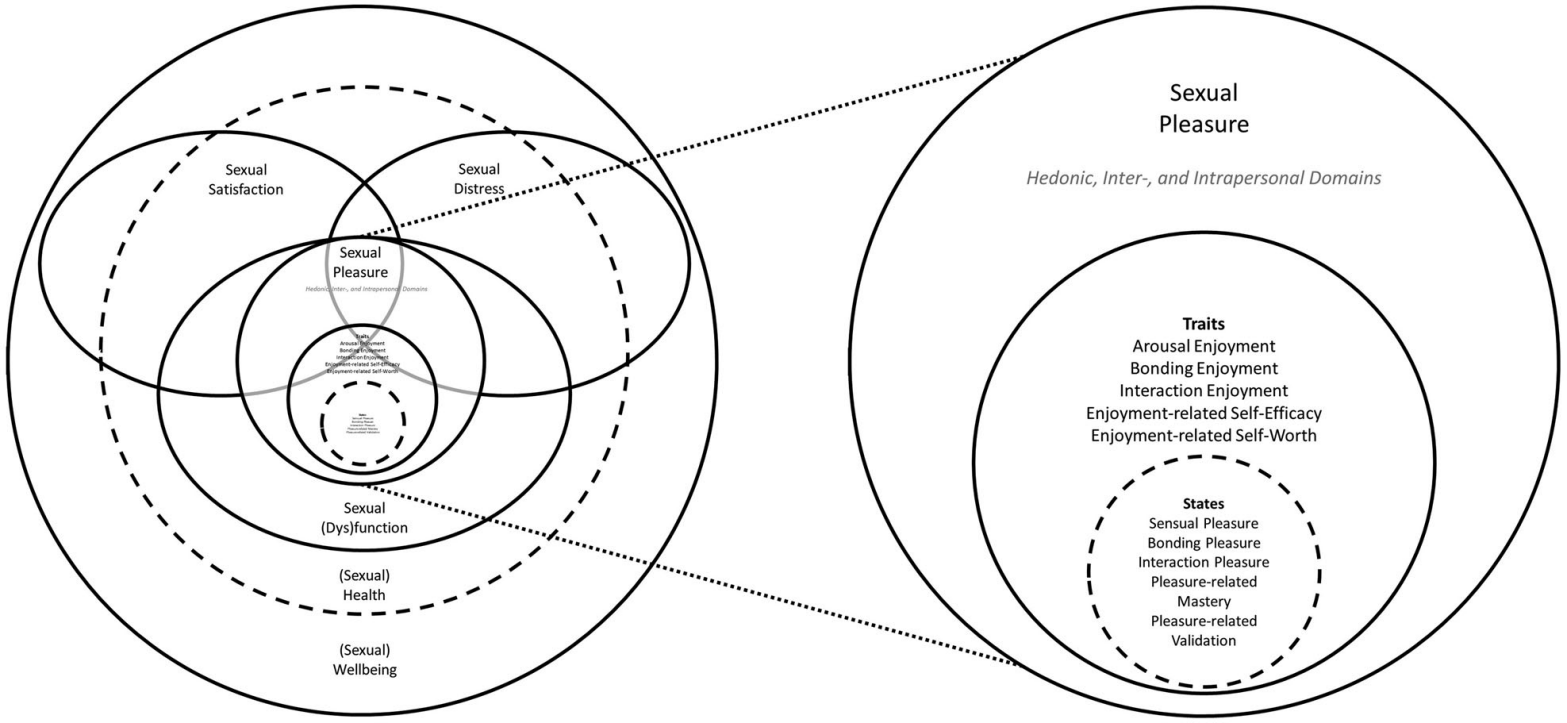
A Diagram of Sexual Pleasure and its Conceptual Relatives. *Note.* The diagram does not specify causal mechanisms but conceptual overlap. We offer this visual alternative to the verbal conceptual specifications in the text.

Finally, sexual pleasure also partakes in sexual wellbeing, with wellbeing encompassing more than sexual pleasure (Fortenberry et al., 2019, Fava & Fortenberry, 2021). According to the GAB definition, sexual pleasure is not synonymous with but contributes to an individual’s (and their partners’) sexual health and wellbeing by means of “key enabling factors”: “Self-determination, consent, safety, privacy, confidence and the ability to communicate and negotiate sexual relations” (GAB, 2016). This suggestion implies that an individual can experience pleasure without such pleasure contributing to their own or their partners’ health and wellbeing. Such “partner/context/event-specific factors” (Fava & Fortenberry, 2021) are not all included in the pleasure-traits of our taxonomy because they are not all part of an individual’s trait repertoire but denote qualities of the sexual situation and interaction (e.g., availability of protection against STIs or unwanted pregnancy and interpersonal safety and privacy), a limitation which we will discuss further in the following section on assessment. We also do not include more distal enabling psychosocial factors for the experience of sexual pleasure, such as a positive body image, which probably facilitate and therefore predict the experience of rewards but should not be equated with it or be included in a pleasure (trait) assessment (see for a review of such predictive factors, Reis et al., 2021 and Fava & Fortenberry, 2021). For a visual summary of the suggested conceptual distinctions, we refer the reader to the diagram in Figure 4.

## Aim 3: Assessing sexual pleasure – How conceptual definitions and operational definitions interrelate

We suggest that an encompassing sexual pleasure assessment battery should assess state sexual pleasure, loose trait sexual pleasure, and strict sexual pleasure traits associated with the different rewards. How we assess sexual pleasure has implications for how the concept can be defined and interpreted (cf. Jackson & Maraun, 1996; Markus, 2008). For instance, assessing differences in sexual pleasure in terms of unifaceted or multifaceted pleasure has implications on where and whether to intervene: is someone satisfied by the presence of or distressed by the absence of sensual pleasure or bonding pleasure? Assessing differences in sexual pleasure in terms of state or trait sexual pleasure has implications for how to intervene: do we need to strengthen strict sexual pleasure capacities or the competence of usually experienced stimulus situations in order to help people experience more state and loose trait sexual pleasure?

The question whether self-report trait-assessments assess traits in the loose sense as tendencies for experience or whether they allow us to infer traits in the strict sense as internal causes of experience has long been discussed by personality psychologists (e.g., DeYoung, 2015; Fleeson, 2001; Mischel, 2009). This discussion about operational definitions relates back to the conceptual question whether behavior in the moment (state) or across moments (loose traits) is a result of the person (traits in the strict sense) or situations (qualities of stimuli) (Fleeson & Noftle, 2008; Mischel, 2009). In our conceptual framework, we follow the interactionist perspectives within the person-situation debate (Schmitt & Blum, 2020), namely that differences in (the tendency to experience) state sexual pleasure (traits in the loose sense) are a function of the inter-individual differences in the capacity to experience sexual pleasure (the person; traits in the strict sense) and differences in the contextual likelihood to encounter rewarding sexual situations (the situation; e.g., qualities of stimuli).

**Figure 5.**
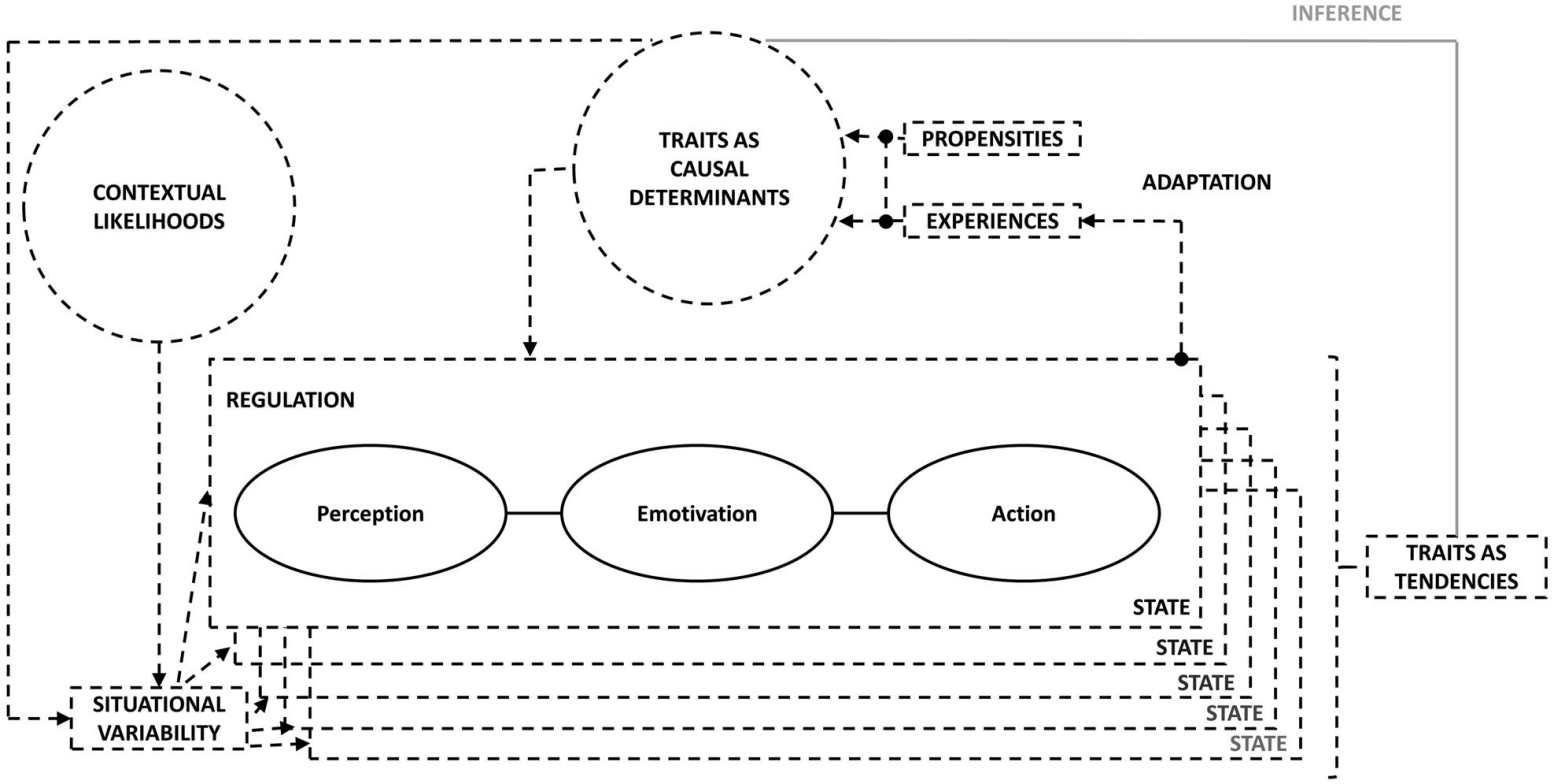
A Diagram of the Interrelationship between States and (Strict and Loose) Traits. *Note.* The diagram is inspired by the work of Fleeson (2001), DeYoung (2015), and Robinaugh et al. (2019). States are represented by each individual box, loose traits are represented as the tendency for experience across several state experiences, and strict traits are represented as causal factors that determine how a state (and thereby loose trait) evolves and regulates. States are further shaped by the situations in which they occur, with situational variability being affected by contextual likelihoods and the traits of the individual. The traits are a function of nature and nurture, with inborn propensities being shaped by experience through adaptation, which is a function of someone’s (state) experiences. Psychometrically, we often infer strict traits from (self-)reports of someone’s tendency for experience, i.e., loose traits. Adaptation occurs through different learning mechanisms, among which, as discussed here, conditioning and heterostasis. Regulation can occur through several mechanisms, among which, as discussed here, physiological homeostasis. Other mechanisms for adaptation and regulation exist, such as, for instance, social modeling (e.g., Bandura, 2005) and emotion regulation strategies (e.g., McRae & Gross, 2020) respectively.

When we accept the premise of the person x situation interaction perspective and want to operationally define all sexual pleasure aspects, we encounter the same old conundrum in the operational definition as in the conceptual definition of states and traits: self-report assessment of state and loose trait sexual pleasure only ever assesses a mixture of strict traits *and* the characteristics of situations (see Figure 5). That is, if we ask individuals “how pleasurable their (last) sexual encounter(s) has(have) been” we cannot infer whether differences between individuals in state sexual pleasure at a time or in loose trait sexual pleasure within individuals over time are due to differences in pleasure traits or differences in the quality of the assessed situations due to differences in contextual likelihoods to encounter rewarding situations. We cannot disentangle variability in state pleasure or its capacities from variability in the quality of situations.

On the one hand, individual differences in strict sexual pleasure traits and state sexual pleasure can only be validly assessed by means of self-report assessments on actual experiences if and only if we can assume that people are interchangeable in the likelihood with which they encounter sexually competent situations or if a presented stimulus situation is equivalent in competence for the people assessed (e.g., Janssen et al., 2003; Wierzba et al., 2015). Also, strict traits might therefore best be assessed via assessments of (cap)abilities rather than self-report, or self-report of usual experience to standardized situations (see the item structure of the SIS/SES; Janssen et al., 2002). On the other hand, a self-report state or loose trait sexual pleasure assessment would purely assess variability in the quality of the sexual situation if participants show no differences in sexual pleasure capacities. Both are usually not the case (Bradford & Spencer, 2020; Higgins et al., 2022; Laan et al., 2021; Sakaluk et al., 2014; Van Anders et al., 2022).

In order to disentangle the variability via self-report, we would need a way to assess differences in the likelihood to encounter rewarding situations. So far, it is impossible to assess the objective reward-level of people’s sexual experiences because there is no way to assess the competence of stimuli independent from the experience of the stimuli – the reward-value of a stimulus is inside the person, not outside of them, and cannot be independently verified. We also do not have an assessment instrument, inter-subjective standard, or norm to quantify and standardize the characteristics of the sexually competent *situation* (see for a discussion of the “psychological” situation, Rauthmann & Sherman, 2020). We therefore cannot tear apart situational and strict trait-related variability and assess how their variability relates to variability in state-pleasure or loose trait-pleasure.

Nevertheless, we would still need a self-report assessment of state and loose trait sexual pleasure to assess these pleasure aspects and to relate them to a (future) stimulus database of normed sexually competent situations or an assessment of situations differing in partner/event/context-related aspects (see for inspiration, Fava & Fortenberry, 2021; Higgins et al., 2022; McClelland, 2010). For instance, we could assess how self-report state-pleasure relates to the kind of stimulation given (for instance, absence or presence of clitoral stimulation) by whom (a fling or a steady partner) in what kind of situation (safe or unsafe) and how such variability compares to variability in the self-reported tendency to experience pleasure. Questions which are worthy to be asked, and possible to be researched by a self-report assessment of state and loose trait sexual pleasure.

Finally, we want to note that self-report is, by definition, a reflection of (recollected) introspection at the conscious level. However, affective processes are not always accessible to introspection, never mind recall of experiences during which such processes took place. Nonconscious state “liking” is assessed differently and it is to future research to see how conscious and nonconscious pleasure might differentially predict and explain sexual experience (see, for instance, recording facial reactions, approach-avoidance tasks, measuring viewing time, implicit association tasks, or measuring hedonic hotspot activation; Berridge & Robinson, 2003; Kringelbach & Berridge, 2010; van Lankveld et al., 2020). Creating a self-report assessment of consciously experienced pleasure allows us to compare it to results using other methodologies. We are working on creating such self-report assessment in future contributions.

## Discussion

In this article, we have suggested that the concept of sexual pleasure can be conceptualized as covering state and (loose and strict) trait components which cover the experience of sexual pleasure and the tendency and capacity to experience sexual pleasure, respectively. These concepts can be used to conceptualize differences in sexual pleasure between people at a moment in time and within people over time, in which state and (loose) trait sexual pleasure are a function of the rewards in a stimulus situation and (strict) sexual pleasure traits. We have argued that state sexual pleasure, as the affective experience of anticipatory and consummatory liking (experience of positive affect, “feeling good”; cf. Smuts, 2011) in response to rewards in a concrete situation at a specific moment in time, should be embedded within the sexual response and its function, but should be distinguished from the other components that make up the sexual response. We have suggested that the experience of liking during sex is not only induced by liking associated with arousal and orgasm, but also liking induced by other rewards which can be served by sexual activity, allowing for a multifaceted perspective on sexual pleasure. Our conceptual synthesis resulted in a taxonomy covering the concepts in Table 2 and Figures 4 and 5. We hereby address the need for a multifaceted perspective on sexual pleasure. To the best of our knowledge, it is the first time that sexual pleasure has been broken down in this way, providing a multifaceted taxonomy and framework of the overall concept.

## Limitations, future outlook, and implications

### WEIRDness of theorists and researchers

The proposed taxonomy relies on theories and research with a western, educated, industrialized, rich, and democratic (WEIRD) background (Henrich et al., 2010). This WEIRD lens extends to our (post)positivist research assumptions, which allow us to pursue a nomological (rather than idiographic) conceptual and operational definition of sexual pleasure, a taxonomy of rewards, and framework based on findings from positivist research methodologies (Steinmetz, 2005). Our assumptions, especially those regarding the existence of certain *universal basic* rewards that are retrieved from and experienced during sexual activity, need to be cross-validated, for instance by means of response-process research and cognitive interviews across different cultural settings in order to validate whether the framework and taxonomy do reflect peoples’ lived experience (Wolf et al., 2019). Smuts (2023), Khalaf et al. (2018), and Muhanguzi (2015) suggest that sensual pleasure is universally experienced but contextually shaped and curtailed by positioning and legitimizing it within the confines of heterosexual, and often married, coupledom and demoralizing it outside these confines. Assessment and research of the sexually pleasurable situation needs to take such aspects into account. Eventually, we attempted to adhere to the GAB definition which states that “[t]he experiences of human sexual pleasure are diverse” by including an array of rewards experienced during sex, rather than equating sexual pleasure with sensual pleasure and orgasm, and by informing our framework and taxonomy by insights gained from positivist and non-positivist research methodologies.

### Aspects in need of clarification

We would like to discuss eight limitations in need of clarification. First, future research needs to ascertain which of the pleasure facets represent useless and potentially misleading reifications, and which facets might require further specification (e.g., whether interaction enjoyment and pleasure comprise reciprocal sharing, i.e., giving *and* receiving, [Opperman et al., 2014; Brown et al., 2018; Lawrance & Byers, 1995] or the giving of pleasure only [Muise & Impett, 2015; Muise et al., 2013]). Second, future research needs to clarify how the domains and facets relate to each other and in what constellation they predict sexual satisfaction, health, and wellbeing in what contexts (Fava & Fortenberry, 2021; GAB, 2016; Gianotten et al., 2021; Kleinplatz et al., 2009; Metz, & McCarthy, 2007). Third, the introduced facets describe categorizations intended to provide an overview, and like any categorization, simplify their content. We simplified the microscopic subtleties of the basic affect accounts and behavioral neuroscience to serve the overall goal of a conceptual, molar-level, synthesis (de la Fuente et al., 2019). Fourth, this limitation includes that a future sexual response framework needs to clarify the interrelationship or overlap between arousal and wanting and incorporate punishment and aversion related processes next to reward. Fifth, we could not yet further clarify the difference in category-non/specificity of genital arousal response between cis women and cis men (see for an excellent review, Chivers, 2017). Sixth, future research needs to assess whether and how the facets vary intra- *and* inter-individually – such variance is not equivalent or interchangeable. Seventh, future work needs to further specify what types of learning, regulation, and adaptation play a role in shaping the sexual (pleasure) response (Berntson & Cacioppo, 2007), a topic we could only shortly touch upon.

Finally, we are presenting a working framework and taxonomy – its content should and needs to be further developed. First, the taxonomy does not yet include “the experience of autonomy” as a reward, nor does it include “enjoyment-related autonomy” as a trait-like pleasure-related capacity or tendency. We suggest that the experience of autonomy seems to be a *contextual predisposition* for pleasure to be experienced and for pleasure to contribute to sexual wellbeing rather than an independent reward (i.e., a “key partner/context/event-specific enabling factors”; GAB, 2016; Fava & Fortenberry, 2021). Second, we did not include “the experience of engagement” as a reward within the taxonomy. We are uncertain whether it should be included as an individual reward or whether this, undeniably pleasurable, state emerges as a potential *consequence* of exhibiting several and/or intense rewards during sex in combination with several enjoyment-traits in an optimal context (i.e., sexual flow, cf. Csikszentmihalyi, 1990; APA, n.d.-f; or sexual peak experience; cf. Privette, 1983; APA, n.d.-e). Third, pleasurable sexual experiences might also have to be experienced as meaningful, as in significant or purposeful, in order to be truly pleasurable (cf. with definitions of the “good life” by e.g., Seligman, 2004 and the definition of optimal or magnificent sex; Kleinplatz et al., 2009; Kleinplatz & Ménard, 2020). We argued that the taxonomy covers pleasurable and potentially engaging experiences, but not whether sexual experiences are experienced as meaningfully “significant”.

### The development of sexual pleasure and its relation to sexual rights

The taxonomy presented here is inspired by learning paradigms which stress the continuous adaptation of an organism to its environment and, thus, the importance of sexual *development* within a person’s learning history alongside human propensities and constraints. Sexual rights start right there; societies should represent environments in which individuals are allowed to sexually flourish by learning to enjoy and desire sex within contexts that respect and stimulate their rights: their individuality, equality and nondiscrimination, psychological and bodily integrity, freedom of opinion and expression, privacy, the highest attainable standard of health, access to education and information and the protection of these rights from infringement by others (Ford et al., 2019; Gruskin et al., 2019). This allows us to envision how we might promote the capacities to experience pleasure and prevent pleasure differentials (Sladden et al., 2021). We might want to foster sexually self-efficacious and self-loving individuals who are allowed to experience sensually pleasurable, safe and secure, validating and engaging, and reciprocal sexual experiences from the start of their sexual interaction careers, such that they develop expectancies that such experiences are attainable and that they can learn how to attain them. Psychology contributes insights that apply to the individual and how individual differences develop within a particular environment, but this is also where the vision of psychology stops. Psychology needs sociology, anthropology, politics, law, philosophy et al. to decide on how interpersonal relationships and the environment might be (re)structured to let the individual flourish, without infringing on other individuals’ flourishing.

## Conclusion

In this article, we have proposed an adapted framework of the sexual response which includes sexual pleasure as a central component. We suggested that state sexual pleasure figures centrally in sexual responding as the experience of anticipatory and consummatory liking (experience of positive affect, “feeling good”; cf. Smuts, 2011) in a concrete situation at a specific moment in time in response to (the expectation of) sexual activity that offers rewards. We have further argued that trait-like concepts can be applied to the tendency to experience such state sexual pleasure which can be used to research differences between people at a moment in time and within people over time. We thereby offered a multifaceted definition of state and trait sexual pleasure and discussed how we might assess pleasure thus defined.

Our multifaceted taxonomy and framework of sexual pleasure might serve as a springboard for clinicians to better understand the diverse factors contributing to patients’ sexual experiences. By considering state and trait sexual pleasure and various rewards, clinicians may find inspiration in developing targeted interventions for specific challenges. However, it is important to approach such developments from the framework with caution, as it requires further direct empirical testing before it can be translated into evidence-based recommendations for clinical practice which can promote fulfilling sexual lives. After the taxonomy’s and a future assessment’s domains and facets are validated, it is to future research to determine how the proposed capacities and qualities of sexual situations might be fostered and whether that prevents individuals from experiencing systematic differences in pleasurable sexual experiences (see for overviews of such systematic differences, Van Anders et al., 2022; Laan et al., 2021; Bradford & Spencer, 2020; Higgins et al., 2022).

Sex and pleasure mean many things to different people (Goldey et al., 2016; Meston & Buss, 2007) and sex can serve pleasure by serving different rewards. We aimed to cover the most relevant in the presented taxonomy and made a valuable conceptual start, worthy of extension. To research and understand sexual pleasure, we need a manner to conceptualize and assess it in all its complexity. By positioning pleasure centrally within the sexual response (system) and offering a first taxonomy and call for a multifaceted assessment of sexual pleasure, we contribute to giving sexual pleasure the center position it deserves in sex research and practice.

## Supplementary Material

Supplemental Material

## Notes

1 The cited affective neuroscience literature is not settled on whether the anticipation of a reward already induces activation of the, so-called, hedonic hotspots that are directly linked to “liking” proper (Berridge, 2018, 2019). Toates (2014, pp. 182–183) argues that pleasure can be experienced during the anticipation of reward (see also, Bindra, 1978). It remains to be seen whether such anticipatory pleasure is associated with hedonic hotspot activation (“liking” proper) or a positive affect process which should be labeled differently, and whether “liking” proper is only associated with consumption rather than anticipation of reward (Berridge, 2018; Paredes, 2014; Pfaus et al., 2012). We therefore decided to use the labels *anticipatory* and *consummatory* liking, both of which should be experienced as pleasure when reaching conscious experience (see also Rømer Thomsen et al., 2015). Eventually, in the case of sexual stimulation and response, we consider it difficult to draw a line between where consumption begins and anticipation stops (Pfaus, 1999). That is, we suggest that affective processes, which are at work during anticipation, flow over into consumption; except if we use the idea of penetration or genital touch defining the line from anticipation to consumption.

2 For some readers, we might gloss over the question whether there is “sexual” versus “general” arousal and/or whether there are different kinds or components of “arousals” (for instance, central, peripheral, or genital arousal; Bancroft, 1989; or wakeful, autonomic, and affective arousal; Satpute et al., 2019). This question is indeed not settled (cf. Ågmo, 2008; Janssen, 2011; Laan et al., 1995; Pfaff, 1999; Sachs, 2007). Here, we follow Ågmo (2008) and others (e.g., Pfaff, 1999) and suggest that there is general, including genital, arousal triggered by environmental changes reflecting potential threats or opportunities that require the mobilization of energy (Frijda, 2008; see also, Russell & Barrett, 1999). Such arousal can be considered “sexual” if exteroceptive or interoceptive stimuli that are associated or experienced concurrently with it, carry non/conscious sexual meanings/connotations and allow the embodied and contextualized experience to be constructed as sexual (Barrett, 2009, 2017; Both et al., 2007). Some readers might consider this a slide of hand and a violation of Occam’s razor, and would argue that we displace the issue of defining what makes certain arousal “sexual” compared to, for instance, “anxious” to a different process – that of construction of basic affective processes. For these readers, we discuss the underlying issue of basic versus constructionist views of emotion in the discussion.

3 This *emergent* state has also been called a “central motive state” (Berridge, 2018; Toates, 2009, 1986) or “presently excited gnostic organization a.k.a. pexgo” (Bindra, 1978). Note also that we are not the first to suggest that the state of experiencing sexual desire is an *emergent* property. Singer and Toates (1987) used the metaphor of a pie emerging from more basic ingredients, and the constructionist perspective on emotions by Barrett (2009) suggests that emotional states metaphorically resemble loafs of bread that emerge from more basic, and interoceptively hardly distinguishable, ingredients.

4 We realize that conceptualizing a strict “trait-like difference” in arousal, wanting, or liking is still rather imprecise. It might help us to label and envision that individuals differ in certain responses amongst each other and amongst themselves across time in response to the same stimulus (situation), but it should be further specified what such differences denote: the relative latency, speed, rate, or absolute intensity with which one responds in response to the stimulus (cf. Pfaus, 1999; Whalen, 1966)? And is that a characteristic of the circuitries or the relationship between the circuitries? And should such molar-level differences between individuals be reduced to microscopic characteristics of individual circuitries at all? Future work needs to answer these questions.

5 We are aware of the circularity in explaining certain stimuli or stimulus situations as rewarding because they trigger pleasure/liking and wanting, and explaining pleasure as a response to rewarding stimuli. However, we suggest that this circularity is not logically problematic as long as we accept the assumption that there are certain stimuli and/or stimulus situations that just are rewarding, i.e., just trigger pleasure/liking and wanting, when they are anticipated, attained, or consumed. Following such a stimulus view of reward does not appear more logically problematic than proposing the existence of an (additional) endogenous “need” mechanism for sexual gratification (“libido”), which causes tension when deprived, and which requires a certain nutrient stimulus in order for the need-tension to be quenched, with quenching the tension equaling pleasure – rather than that the contact with the rewarding stimulus itself is what is pleasurable. The existence of a “need” is not necessary to explain reward, contact with rewarding stimuli and processes mediating reward are (see Hilgard and Marquis, 1961b).
