# Supplementary material for: Sexual Pleasure Matters – and How to Define and Assess It Too. A Conceptual Framework of Sexual Pleasure and the Sexual Response
Source: Int J Sex Health. 2023 Jun 12;35(3):313–40. doi: 10.1080/19317611.2023.2212663 (PMC10903685; doi:10.1080/19317611.2023.2212663)
Supplement: Supplemental Material [file WIJS_A_2212663_SM7329.docx]

**Supplementary Material**

**Sexual Pleasure Matters – and How to Define and Assess It Too.**

**A Conceptual Framework of Sexual Pleasure and the Sexual Response.**

**Definitions of Sexual Pleasure - Reviewed Articles**

Some of the cited articles demarcate sexual pleasure from, for instance, orgasmic experience; however, they do not propose a definition of sexual pleasure following that demarcation. Also, some studies are qualitative studies which, by design, leave definitions to the participants of the study. We did include those qualitative studies in Table 1 in the main text if the study proposed different components of pleasure and respective labels or definitions.

Note that the articles were not retrieved via a systematic search; however, we reviewed studies included in systematic reviews (e.g., Reis et al., 2021), in addition to the articles we had read in preparation for the main article. We therefore assume that the selection includes a representative overview of the available literature on definitions and assessments of sexual pleasure.

**(1) No definition cited nor proposed:** Thirlaway et al. (1996), Slosarz (2000), Ferguson et al. (2003), Pinkerton et al., (2003), Sanchez et al. (2005), Sanchez and Kiefer (2007), Randolph et al., (2007), Higgins et al., (2008), Hull (2008), Boul et al. (2008), Maynard et al., (2009), Vannier and O'Sullivan (2010), Hinchliff et al. (2010), Schick et al. (2010), Weinberg and Williams (2010), Herbenick et al., (2011), Kaestle and Allen (2011), Backstrom et al (2012), Lorvick et al. (2012), Tambling et al., (2012), Kisa and Özdemir (2013), Stulhofer and Ajduvić (2013), Fahs (2014), Bowman (2014), Fahs and Gonzalez (2014), Fennell (2014), Herbenick et al., (2014), Opperman et al., (2014), Smith et al. (2014), Fileborn et al., (2015), Hoel (2015), Mastro and Zimmer-Gembeck (2015), John et al. (2015), Barnett and Melugin (2016), Shepardson et al., (2016), Wood et al., (2016), Thomas et al. (2017), Blunt-Vinti et al. (2018), Brown et al. (2018), Grower and Ward (2018), Herbenick et al., (2018), Khalaf et al., (2018), Rigo and Saroglou (2018), Siegler et al., (2018), Alarie (2019), Ashton et al. (2019), Benson et al. (2019), Stahl et al., (2019), Carter et al. (2019), Chadwick et al. (2019), Guitelman et al., (2019), Herbenick et al., (2019), Katzman and Tuchman (2019), Marques (2019), Muhanguzi (2015), Thomas and Copulsky (2020), Waskul and Anklan (2020), Weitkamp et al. (2020), Pascoe (2021).

**(2) Philpott et al. (2006)/GAB (2016) definition:** Fiaveh et al. (2015), Pascoal et al., (2016), Saliares et al., (2017), De Santis et al., (2019), Ford et al. (2019), Castellanos-Usigli and Braeken-van Schaik (2019), and Gruskin et al., (2019), Boydell et al. (2021).

**(3) Abramson and Pinkerton (2002) definition:** Rye and Meaney (2007), Hargons et al. (2018), Bowling et al. (2018).

**(4) Pleasure as “physical sensation(s)”:** Zurbriggen and Yost (2004)**,** Rowland et al., (2019).

**References**

Abramson, P. R., & Pinkerton, S. D. (2002). *With pleasure: Thoughts on the nature of human sexuality.* New York: Oxford University Press.

Alarie, M. (2019). Sleeping with younger men: Women’s accounts of sexual interplay in age-hypogamous intimate relationships. *The Journal of Sex Research, 57*(3), 322-334.

Ashton, S., McDonald, K., & Kirkman, M. (2019). Pornography and women’s sexual pleasure: Accounts from young women in Australia. *Feminism & Psychology, 29*(3), 409-432.

Backstrom, L., Armstrong, E. A., & Puentes, J. (2012). Women's negotiation of cunnilingus in college hookups and relationships. *Journal of Sex Research, 49*(1), 1-12.

Barnett, M. D., & Melugin, P. R. (2016). Reported sexual pleasure among heterosexual men and women: An empirical investigation. *Personality and Individual Differences, 98*, 62-68.

Benson, L. S., Gilmore, K. C., Micks, E. A., McCoy, E., & Prager, S. W. (2019). Perceptions of anal intercourse among heterosexual women: A pilot qualitative study. *Sexual Medicine, 7*(2), 198-206.

Blunt-Vinti, H. D., Stokowski, S. E., & Bouza, B. M. (2018). “Your vagina is not supposed to be this scary monster”: Young heterosexual women’s recommendations for improving sexual satisfaction and implications for sexuality education. *American Journal of Sexuality Education, 13*(2), 245-265.

Boul, L., Hallam-Jones, R., & Wylie, K. R. (2008). Sexual pleasure and motivation. *Journal of Sex & Marital Therapy, 35*(1), 25-39.

Bowling, J., Dodge, B., Bindra, N., Dave, B., Sharma, R., Sundarraman, V., Dharuman, S. T. & Herbenick, D. (2018). Female condom acceptability in urban India: Examining the role of sexual pleasure. *Journal of Health Psychology, 23*(2), 218-228.

Bowman, C. P. (2014). Women’s masturbation: Experiences of sexual empowerment in a primarily sex-positive sample. *Psychology of Women Quarterly, 38*(3), 363-378.

Boydell, V., Wright, K. Q., & Smith, R. D. (2021). A Rapid Review of Sexual Pleasure in First Sexual Experience(s), *The Journal of Sex Research*.

Brown, J., Schmidt, J., & Robertson, N. (2018). “We’re like the sex CPR dummies”: Young women’s understandings of (hetero) sexual pleasure in university accommodation. *Feminism & Psychology, 28*(2), 253-271.

Carter, A., Ford, J. V., Luetke, M., Fu, T. C. J., Townes, A., Hensel, D. J., Dodge, B. & Herbenick, D. (2019). “Fulfilling His Needs, Not Mine”: Reasons for not talking about painful sex and associations with lack of pleasure in a nationally representative sample of women in the United States. *The Journal of Sexual Medicine, 16*(12), 1953-1965.

Castellanos-Usigli, A., & Braeken-van Schaik, D. (2019). The pleasuremeter: Exploring the links between sexual health, sexual rights and sexual pleasure in sexual history-taking, SRHR counselling and education. *Sexual and Reproductive Health Matters, 27*(1), 313-315.

Chadwick, S. B., Francisco, M., & van Anders, S. M. (2019). When orgasms do not equal pleasure: Accounts of “bad” orgasm experiences during consensual sexual encounters. *Archives of Sexual Behavior, 48*(8), 2435-2459.

De Santis, C., Murray, S. H., Kohut, T. & Milhausen, R. R. (2019). Good gets better? Examining the impact of prescribed sexual activity on long-term heterosexually configured couples’ sexual and relationship satisfaction, *Sexual and Relationship Therapy, 34*(2), 173-192.

Fahs, B. (2014). Coming to power: Women’s fake orgasms and best orgasm experiences illuminate the failures of (hetero)sex and the pleasures of connection. *Culture, Health & Sexuality, 16*(8), 974-988.

Fahs, B., & Gonzalez, J. (2014). The front lines of the “back door”: Navigating (dis)engagement, coercion, and pleasure in women’s anal sex experiences. *Feminism & Psychology, 24*(4), 500-520.

Fennell, J. (2014). “And Isn't that the point?”: Pleasure and contraceptive decisions. *Contraception, 89*(4), 264-270.

Ferguson, D., Steidle, C., Singh, G., Alexander, J., Weihmiller, M., & Crosby, M. (2003). Randomized, placebo-controlled, double blind, crossover design trial of the efficacy and safety of Zestra for women in women with and without female sexual arousal disorder. *Journal of Sex & Marital Therapy, 29*, 33-44.

Fiaveh, D. Y., Okyerefo, M. P., & Fayorsey, C. K. (2015). Women’s experiences of sexual pleasure in Ghana. *Sexuality & Culture, 19*(4), 697–714.

Fileborn, B., Thorpe, R., Hawkes, G., Minichiello, V., Pitts, M., & Dune, T. (2015). Sex, desire and pleasure: Considering the experiences of older Australian women. *Sexual and Relationship Therapy, 30*(1), 117-130.

Ford, J. V., Corona Vargas, E., Finotelli Jr, I., Fortenberry, J. D., Kismödi, E., Philpott, A., Rubio-Aurioles, E., & Coleman, E. (2019). Why pleasure matters: Its global relevance for sexual health, sexual rights and wellbeing. *International Journal of Sexual Health, 31*(3), 217-230.

GAB. (2016). Working definition of Sexual Pleasure. Retrieved from https://www.gab-shw.org/our-work/working-definition-of-sexual-pleasure/

Grower, P., & Ward, L. M. (2018). Examining the unique contribution of body appreciation to heterosexual women’s sexual agency. *Body Image, 27*, 138-147.

Gruskin, S., Yadav, V., Castellanos-Usigli, A., Khizanishvili, G., & Kismödi, E. (2019). Sexual health, sexual rights and sexual pleasure: Meaningfully engaging the perfect triangle. *Sexual and Reproductive Health Matters, 27*(1), 29-40.

Guitelman, J., Mahar, E. A., Mintz, L. B., & Dodd, H. E. (2019). Effectiveness of a bibliotherapy intervention for young adult women’s sexual functioning. *Sexual and Relationship Therapy,* 1-21.

Hargons, C. N., Mosley, D. V., Meiller, C., Stuck, J., Kirkpatrick, B., Adams, C., & Angyal, B. (2018). “It feels so good”: Pleasure in last sexual encounter narratives of black university students. *Journal of Black Psychology, 44*(2), 103–127.

Herbenick, D., Fu, T. C., Arter, J., Sanders, S. A., & Dodge, B. (2018). Women's experiences with genital touching, sexual pleasure, and orgasm: Results from a US probability sample of women ages 18 to 94. *Journal of Sex & Marital Therapy, 44*(2), 201-212.

Herbenick, D., Fu, T. C., Dodge, B., & Fortenberry, J. D. (2019). The alcohol contexts of consent, wanted sex, sexual pleasure, and sexual assault: Results from a probability survey of undergraduate students. *Journal of American College Health, 67*(2), 144-152.

Herbenick, D., Reece, M., Hensel, D., Sanders, S., Jozkowski, K., & Fortenberry, J. D. (2011). Association of lubricant use with women's sexual pleasure, sexual satisfaction, and genital symptoms: A prospective daily diary study. *The Journal of Sexual Medicine, 8*(1), 202-212.

Herbenick, D., Reece, M., Schick, V., Sanders, S. A., & Fortenberry, J. D. (2014). Women's use and perceptions of commercial lubricants: Prevalence and characteristics in a nationally representative sample of American adults. *The Journal of Sexual Medicine, 11*(3), 642-652.

Higgins, J. A., Hoffman, S., Graham, C. A., & Sanders, S. A. (2008). Relationships between condoms, hormonal methods, and sexual pleasure and satisfaction: An exploratory analysis from the women’s well-Being and sexuality study. *Sexual Health, 5*(4), 321-330.

Hinchliff, S., Gott, M., & Ingleton, C. (2010). Sex, menopause and social context: A qualitative study with heterosexual women. *Journal of Health Psychology, 15*(5), 724-733.

Hoel, N. (2015). Engaging Islamic sexual ethics: Intimacy, pleasure and sacrality. *Sexualities, 18*(1-2), 80-99.

Hull, T. H. (2008). Sexual pleasure and wellbeing. *International Journal of Sexual Health, 20*(1-2), 133-145.

John, N. A., Babalola, S., & Chipeta, E. (2015). Sexual pleasure, partner dynamics and contraceptive use in Malawi. *International Perspectives on Sexual and Reproductive Health, 41*(2), 99-107.

Kaestle, C. E., & Allen, K. R. (2011). The role of masturbation in healthy sexual development: Perceptions of young adults. *Archives of Sexual Behavior, 40*(5), 983-994.

Katzman, C., & Tuchman, E. (2019). The intersection of women’s sexual pleasure and injection drug use. *Substance Abuse, 40*(2), 201-206.

Khalaf, Z. F., Liow, J. W., Low, W. Y., Ghorbani, B., & Merghati-Khoei, E. (2018). Young women’s experience of sexuality: A battle of pleasure and sexual purity in the context of Malaysian society. *Sexuality & Culture, 22*(3), 849-864.

Kisa, S., & Özdemir, N. (2013). Vaginal dryness problems among southeastern women aged 40-65 years in Turkey. *Sexuality and Disability, 31*(1), 71-82.

Lorvick, J., Bourgois, P., Wenger, L. D., Arreola, S. G., Lutnick, A., Wechsberg, W. M., & Kral, A. H. (2012). Sexual pleasure and sexual risk among women who use methamphetamine: A mixed methods study. *International Journal of Drug Policy, 23*(5), 385-392.

Marques, O. (2019). Navigating, challenging, and contesting normative gendered discourses surrounding women’s pornography use. *Journal of Gender Studies, 28*(5), 578-590.

Mastro, S., & Zimmer-Gembeck, M. J. (2015). Let's talk openly about sex: Sexual communication, self-esteem and efficacy as correlates of sexual well-being. *European* *Journal of Developmental Psychology, 12*(5), 579-598.

Maynard, E., Carballo‐Diéguez, A., Ventuneac, A., Exner, T., & Mayer, K. (2009). Women's experiences with anal sex: Motivations and implications for STD prevention. *Perspectives on Sexual and Reproductive Health, 41*(3), 142-149.

Muhanguzi, F. K. (2015). “Sex is sweet”: women from low-income contexts in Uganda talk about sexual desire and pleasure. *Reproductive Health Matters, 23*(46), 62-70.

Opperman, E., Braun, V., Clarke, V., & Rogers, C. (2014). “It feels so good it almost hurts”: Young adults' experiences of orgasm and sexual pleasure. *The Journal of Sex Research, 51*(5), 503-515.

Pascoal, P. M., Sanchez, D. T., Raposo, C. F., & Pechorro, P. (2016). Initial validation of the Sexual Pleasure Scale in clinical and non-clinical samples of partnered heterosexual people. *The Journal of Sexual Medicine, 13*(9), 1408-1413.

Pascoe, L. (2021). Negotiating HIV and pregnancy prevention and sexual pleasure amongst heterosexual men and women in South Africa. *Culture, Health & Sexuality, 23*(1), 115-130.

Philpott, A., Knerr, W., & Boydell, V. (2006). Pleasure and prevention: When good sex is safer sex. *Reproductive Health Matters, 14*(28), 23-31.

Pinkerton, S., Cecil, H., Bogart, L., & Abramson, P. (2003). The pleasures of sex: An empirical investigation. *Cognition & Emotion, 17*(2), 341-353.

Randolph, M. E., Pinkerton, S. D., Bogart, L. M., Cecil, H., & Abramson, P. R. (2007). Sexual pleasure and condom use. *Archives of sexual behavior, 36*(6), 844-848.

Reis, J., de Oliveira, L., Oliveira, C., & Nobre, P. (2021). Psychosocial and Behavioral Aspects of Women’s Sexual Pleasure: A Scoping Review. *International Journal of Sexual Health,* 1-22.

Rigo, C., & Saroglou, V. (2018). Religiosity and sexual behavior: Tense relationships and underlying affects and cognitions in samples of Christian and Muslim traditions. *Archive for the Psychology of Religion, 40*(2-3), 176-201.

Rowland, D., Donarski, A., Graves, V., Caldwell, C., Hevesi, B., & Hevesi, K. (2019). The experience of orgasmic pleasure during partnered and masturbatory sex in women with and without orgasmic difficulty. *Journal of Sex & Marital therapy, 45*(6), 550-561.

Rye, B., & Meaney, G. J. (2007). The pursuit of sexual pleasure. *Sexuality & Culture, 11*(1), 28-51.

Saliares, E., Wilkerson, J. M., Sieving, R. E., & Brady, S. S. (2017). Sexually experienced adolescents’ thoughts about sexual pleasure. *The Journal of Sex Research, 54*(4-5), 604-618.

Sanchez, D. T., Crocker, J., & Boike, K. R. (2005). Doing gender in the bedroom: Investing in gender norms and the sexual experience. *Personality and Social Psychology Bulletin, 31*(10), 1445-1455.

Sanchez, D. T., & Kiefer, A. K. (2007). Body concerns in and out of the bedroom: Implications for sexual pleasure and problems. *Archives of Sexual Behavior, 36*(6), 808-820.

Schick, V., Herbenick, D., Reece, M., Sanders, S. A., Dodge, B., Middlestadt, S. E., & Fortenberry, J. D. (2010). Sexual behaviors, condom use, and sexual health of Americans over 50: Implications for sexual health promotion for older adults. *The Journal of Sexual Medicine, 7*, 315-329.

Shepardson, R. L., Walsh, J. L., Carey, K. B., & Carey, M. P. (2016). Benefits of hooking up: Self-reports from first-year college women. *International Journal of Sexual Health, 28*(3), 216-220.

Siegler, A. J., Boos, E., Rosenberg, E. S., Cecil, M. P., & Sullivan, P. S. (2018). Validation of an Event-Level, Male Sexual Pleasure Scale (EMSEXpleasure) among condom-using men in the US. *Archives of sexual behavior, 47*(6), 1745-1754.

Slosarz, W. (2000). Frequency of sexual behaviour and associated pleasure in a population of married Polish students. *Sexual and Relationship Therapy, 15*(1), 59-65.

Smith, N. K., Jozkowski, K. N., & Sanders, S. A. (2014). Hormonal contraception and female pain, orgasm and sexual pleasure. *The Journal of Sexual Medicine, 11*(2), 462-470.

Stahl, K. A. M., Gale, J., Lewis, D. C., & Kleiber, D. (2019). Pathways to pleasure: Older adult women’s reflections on being sexual beings. *Journal of Women & Aging, 31*(1), 30-48.

Stulhofer, A., & Ajduković, D. (2013). A mixed-methods exploration of women’s experiences of anal intercourse: Meanings related to pain and pleasure. *Archives of Sexual Behavior, 42*(6), 1053-1062.

Tambling, R. B., Neustifter, R., Muska, C., Reckert, A., & Rua, S. (2012). Pleasure-centered educational program: A comprehensive approach to pleasure-oriented sexuality education in domestic violence shelters. *International Journal of Sexual Health, 24*(4), 267-289.

Thirlaway, K., Fallowfield, L. & Cuzick, J. (1996). The Sexual Activity Questionnaire: A measure of women's sexual functioning. *Qual Life Res, 5*, 81-90.

Thomas, E. J., Stelzl, M., & Lafrance, M. N. (2017). Faking to finish: Women’s accounts of feigning sexual pleasure to end unwanted sex. *Sexualities, 20*(3), 281-301.

Thomas, J. N., & Copulsky, D. (2020). Diversifying Conceptions of sexual pleasure in self-reported genital piercing stories. *Deviant Behavior,* 1-11.

Vannier, S. A., & O'Sullivan, L. F. (2010). Sex without desire: Characteristics of occasions of sexual compliance in young adults' committed relationships. *Journal of Sex Research, 47*(5), 429-439.

Waskul, D., & Anklan, M. (2020). “Best invention, second to the dishwasher”: Vibrators and sexual pleasure. *Sexualities, 23*(5-6), 849-875.

Weinberg, M. S., & Williams, C. J. (2010). Bare bodies: Nudity, gender, and the looking glass body. *Sociological Forum, 25*(1), 47-67.

Weitkamp, K., Hänisch, I., & Heesch, S. C. (2020). A controlled pilot study to test the online intervention Self: Cervix focusing on cervical pain, numbness, sexual pleasure and well-being. *Psychology & Sexuality*, 1-10.

Wood, J. R., McKay, A., Komarnicky, T., & Milhausen, R. R. (2016). Was it good for you too?: An analysis of gender differences in oral sex practices and pleasure ratings among heterosexual Canadian university students. *The Canadian Journal of Human Sexuality, 25*(1), 21-29.

Zurbriggen, E. L., & Yost, M. R. (2004). Power, desire, and pleasure in sexual fantasies. *Journal of Sex Research, 41*(3), 288-300.
